# Non-equilibrium Steady States in Catalysis, Molecular Motors, and Supramolecular Materials: Why Networks and Language Matter

**DOI:** 10.1021/jacs.2c12665

**Published:** 2023-06-21

**Authors:** Ivan Aprahamian, Stephen M. Goldup

**Affiliations:** † Burke Laboratory, Department of Chemistry, Dartmouth College, Hanover, New Hampshire 03755, USA; ‡ School of Chemistry, University of Birmingham, Birmingham B15 2TT, U.K.

## Abstract

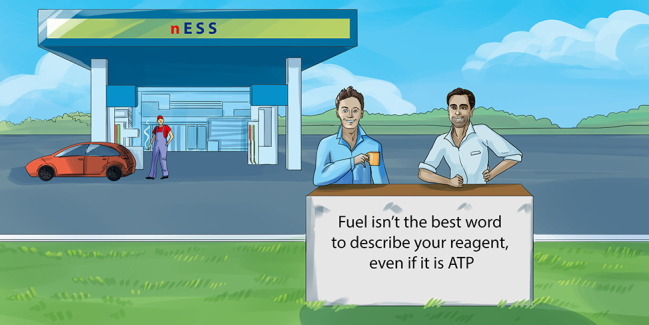

All chemists are familiar with the idea that, at equilibrium steady state, the relative concentrations of species present in a system are predicted by the corresponding equilibrium constants, which are related to the free energy differences between the system components. There is also no net flux between species, no matter how complicated the reaction network. Achieving and harnessing non-equilibrium steady states, by coupling a reaction network to a second spontaneous chemical process, has been the subject of work in several disciplines, including the operation of molecular motors, the assembly of supramolecular materials, and strategies in enantioselective catalysis. We juxtapose these linked fields to highlight their common features and challenges as well as some common misconceptions that may be serving to stymie progress.

## Introduction

Chemists are taught that if molecules **A** and **B** are in dynamic exchange and at equilibrium, there is no net exchange (flux) between them:^1^ every molecule of **A** that is converted to **B** per unit time is balanced by a molecule of **B** converted to **A**. This observation is then used to derive relationships between the rate constants of the exchange, the associated free energy change of converting **A** to **B**, and the corresponding equilibrium constant.^2,3^ Although not always explicitly highlighted, this is a consequence of microscopic reversibility^4^—the transition state for the forward reaction is also the lowest energy pathway for the reverse. These ideas extend to linked equilibria; if **A** is in equilibrium with **B** which is in equilibrium with **C**, the concentrations [**A**], [**B**], and [**C**] satisfy the equilibrium constants *K*_AB_, *K*_BC_, and *K*_AC_.

**Figure 1:**
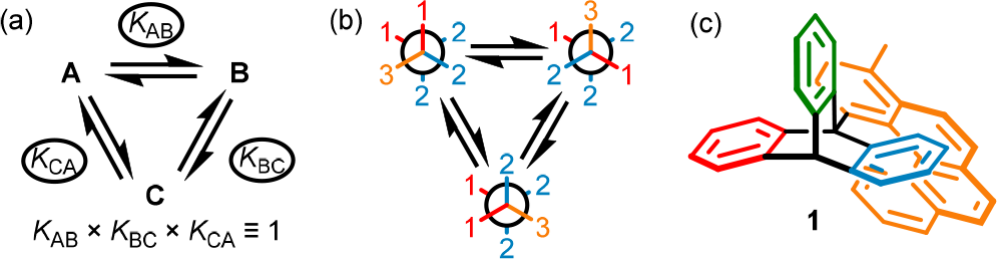
(a) A cyclic equilibrium between **A**, **B**, and **C**. (b) A chemical example of a cyclic equilibrium. (c) Kelly’s helicene rotor **1**(6) with a ratchet-like potential-energy surface that nonetheless fails to rotate unidirectionally at equilibrium.

The same principles extend to cyclic exchange processes (Figure 1a): at equilibrium steady state, there is no net flux around the cycle and all individual equilibrium constants are satisfied. More generally, if multiple pathways connect species in a reaction network, at equilibrium steady state there is no net flux over any single path, which is the condition of “detailed balance”.^3,5^ A simple example is the rotation about a C–C single bond connecting stereogenic and prochiral centers (Figure 1b). Detailed balance holds even when the potential energy surface for the rotation resembles a macroscopic ratchet, as shown by Kelly,^6^ who demonstrated that rotation about the triptycene–helicene bond of **1** (Figure 1c) occurs at equal rates in both directions; if it did not, it would be possible to use flux around the cycle to perform work at equilibrium steady state, violating the second law of thermodynamics.

Less commonly appreciated is the possibility of achieving a non-equilibrium steady state (**NESS**) by coupling^7^ a reaction network to a second spontaneous process. If the components of the coupled reaction are “chemostated” away from their equilibrium concentrations, the concentrations of species in the network may be perturbed from the values predicted by the corresponding equilibrium constants and flux can take place through the network, which can, in principle, be harnessed to do work.

Here we examine systems that achieve a NESS in catalysis, autonomous molecular motors,^8^ and supramolecular materials, with a focus on the requirements that must be met. Reviews on these topics have appeared in recent years that provide varying levels of mathematical detail and typically focus on one area,^9−11^ although examples that discuss self-assembly alongside molecular motors have appeared.^12^ This Perspective is differentiated by an attempt to juxtapose a small number of examples of all three systems with a focus on the role of the coupled reaction and the form of the reaction network. In doing so we are recapitulating the seminal work of Astumian,^13^ who advocates an elegant, quantitative, thermodynamically consistent approach to molecular motors, that was brought into the chemical mainstream by Kay, Leigh, and Zerbetto.^14^ We present quantitative aspects of these systems in a “chemist friendly” manner, but the use of mathematical equations is kept to a minimum; the interested reader will be directed to the Supporting Information (SI), which contains full derivations and additional discussion. Although articles providing different perspectives on the operation of systems at NESS,^15^ we prefer Astumian’s simple and complete approach,^16^ which can be used to recover most if not all the same insights.

Finally, our intention is not only to spur development in these related areas by highlighting their common features and challenges but also to suggest that the language currently used to describe them, which is still in development, is sometimes, in our view, actively unhelpful. Thus, we dedicate a section to the discussion of terminology, and throughout we will avoid using “fuel”, “dissipative”, and “out of equilibrium”. We note that photochemical processes are not required to conform to microscopic reversibility and, hence, do not have the same rigid requirements as chemical equilibria.^17^ Nonetheless, some similar considerations should be applied when examining and designing chemical networks coupled to photochemical processes, which will be commented on at the end of this article. We begin by developing the general quantitative features of reaction networks coupled to a chemostated spontaneous process using a simple example. This section can be skipped if the reader prefers as the key conclusions are reiterated in the subsequent discussion.

## Quantitative Descriptions of Simple Reaction Networks

### 1 Perturbation of Chemical Exchange through Coupling to a Chemostated Spontaneous Process (SI section 1.1)

**Figure 2:**
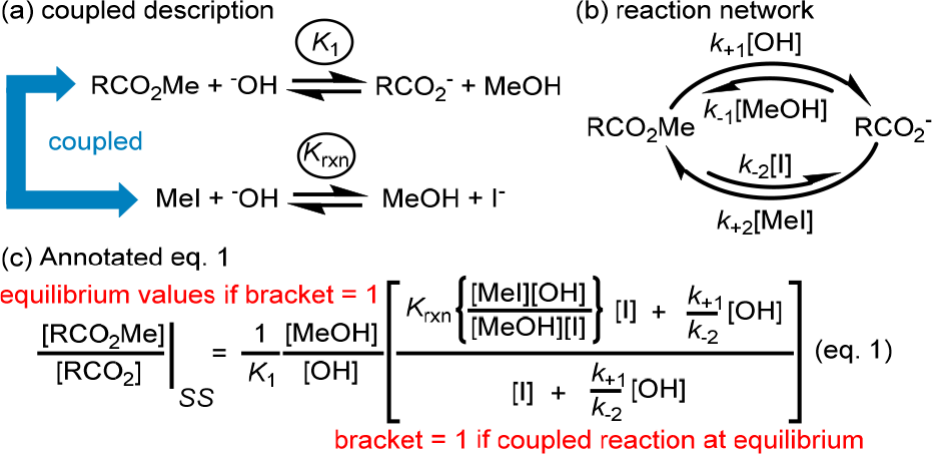
Coupling MeI and ester hydrolysis. (a) The reactions that are coupled. (b) The reaction network established. (c) Annotated equation for the ratio of [RCO_2_Me] and [RCO_2_] at steady state (SS) (charges omitted in concentrations for clarity).

Using Astumian’s trajectory thermodynamics approach^13e^ it is possible to formally demonstrate that “coupling” a simple chemical exchange **A⇌B**, to a general spontaneous process can lead to a NESS in which concentrations [**A**] and [**B**] do not conform to *K*_AB_. For a real system, we can consider the exchange between RCO_2_Me and RCO_2_^–^ coupled to the alkaline hydrolysis of MeI (Figure 2a). For simplicity, we consider the outcome if [MeI], [OH], [MeOH], and [I] (the components of the MeI hydrolysis reaction) are held at fixed values (chemostated). Trajectory thermodynamics can be used to generate an expression (Figure 2c, eq 1) for the relationship between [RCO_2_Me] and [RCO_2_]^18^ at steady state once chemically unphysical steps are discounted. The same expression is reached if we consider a network that includes all of the processes that exchange RCO_2_^–^ and RCO_2_Me (Figure 2b).

The form of the network and eq 1 allow us to make some general observations:

(i) The relative concentrations of [RCO_2_Me] and [RCO_2_] are perturbed from their equilibrium values (i.e., a NESS is achieved) if the coupled reaction is spontaneous *in either direction*.
(ii) Coupling arises practically because the carboxylate anion mediates the hydrolysis of MeI.
(iii) If, as here, we assume an elementary (S_N_2) ester hydrolysis mechanism (formally B_Al_2), there is no net flux between RCO_2_Me and RCO_2_^–^ at NESS, although there is net flux over the two pathways that connect them. If instead hydrolysis proceeds via an intermediate (e.g., B_AC_2), the equations have the same form but in this case there is net flux between all species in the cycle.^19^

### 2 A Network in Which Some Components Are Coupled to a Chemostated Spontaneous Process (SI section 1.2)

**Figure 3:**
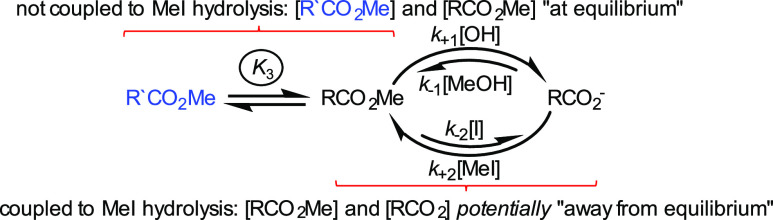
A “linear” network in which RCO_2_Me and R′CO_2_Me are in direct conformational exchange, but only RCO_2_Me is in exchange with RCO_2_^–^.

This simple system can be extended by considering the outcome if the ester can adopt two distinct conformations, RCO_2_Me and R′CO_2_Me, that are in direct unimolecular exchange, only one of which is in direct exchange with RCO_2_^–^ (Figure 3). Although this network appears more complicated, it can readily be shown that the equation relating [RCO_2_Me] and [RCO_2_] is identical to eq 1, whereas the ratio of [RCO_2_Me] to [R′CO_2_Me] is as predicted by the corresponding equilibrium constant.

This leads to the following general observations:

(i) The relative concentrations of species *within* a cycle coupled to a spontaneous process can be perturbed from their equilibrium values, and net flux can take place across the different pathways that connect them.
(ii) The relative concentrations of species not within a coupled cycle are unaffected by the coupled reaction.
(iii) Based on (i) and (ii), crudely speaking, the species within the coupled cycle can be “away from equilibrium” but off-cycle processes must be “at equilibrium”; it is not useful to describe the whole system as “out of equilibrium”.

### 3 A Network in Which All Species Are Coupled to a Chemostated Spontaneous Process (SI Section 1.3)

**Figure 4:**
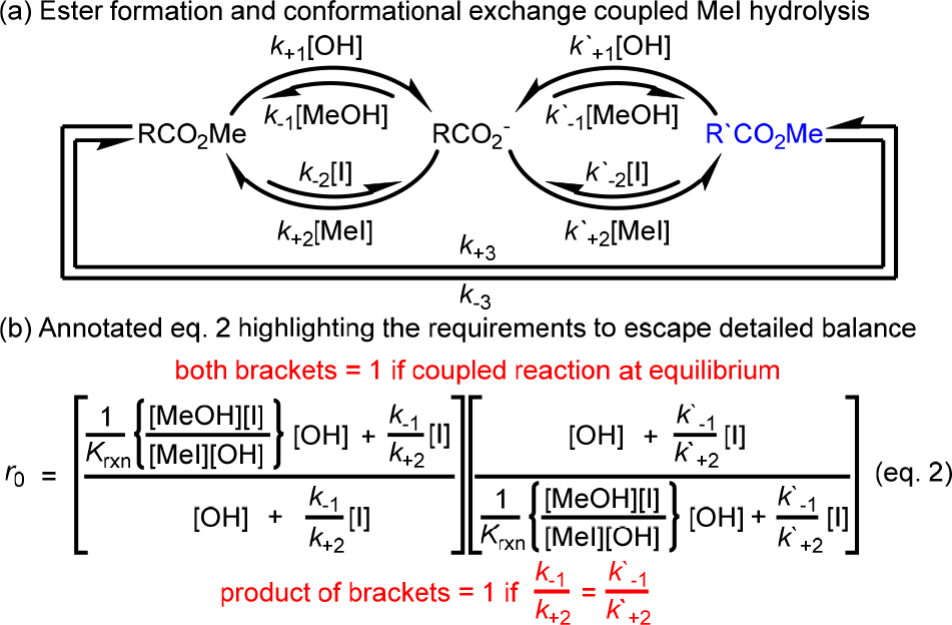
(a) A network in which ester formation/hydrolysis, and conformational exchange between RCO_2_Me and R′CO_2_Me are coupled to MeI hydrolysis. (b) Annotated equation for the ratcheting constant, *r*_0_.

This system can be extended to a cyclic reaction network by considering the outcome if both RCO_2_Me and R′CO_2_Me are in direct exchange with RCO_2_^–^ (Figure 4a). Simple analysis allows us to generate expressions for the relative concentrations of the species involved and the ratcheting constant, *r*_0_ (Figure 4b, eq 2), whose value quantifies flux in the network;^13f^ if *r*_0_ = 1 there is no net flux, whereas if it deviates from 1 there is net flux around the cycle RCO_2_Me → RCO_2_^–^ → R′CO_2_Me at NESS.

The form of eq 2 yields some general observations:

(i) *r*_0_ will always equal 1 (no net flux) if the coupled process (here MeI hydrolysis) is not spontaneous.
(ii) Less intuitively, the network must display “kinetic asymmetry”:^13f^*r*_0_ = 1 if .
(iii) If the coupled reaction is spontaneous *and* the network displays kinetic asymmetry, there will be net flux at steady state (*r*_0_ ≠ 1) and the relative concentrations of the species may be perturbed.
(iv) *r*_0_ depends on the direction of the coupled process; flux can occur in either direction for given rate constants.

## Non-equilibrium Steady States—The Basics

Before discussing more complicated systems, it is essential that the general requirements for and consequences of achieving a NESS are clear. In Quantitative Descriptions of Simple Reaction Networks, we develop the universal features of such systems using a simple chemical example and mathematical equations. The key conclusions are as follows:

(i) For a reaction network to achieve a NESS it must be coupled to a spontaneous process (i.e., the relative concentrations of the species involved in the coupled process do not accord with the corresponding equilibrium constant, here *K*_rxn_ throughout).^20^
(ii) Coupling between the network and the spontaneous process requires species in the former to mediate the latter.
(iii) Even if conditions (i) and (ii) are met, the relative concentrations of species in the network will not be perturbed unless the network displays kinetic asymmetry.^13f^
(iv) At NESS, the relative concentrations of species in the portion of the network coupled to the spontaneous process can be perturbed from their equilibrium values and net flux can take place between them, *but* species outside the portion of the network coupled to the spontaneous reaction are unaffected.^21^

If conditions (i)–(iii) are met, net flux between species in the coupled cycle can be harnessed to do continuous work at NESS. Point (iv) highlights that it is not informative to describe the whole system as “out of equilibrium”; only some parts of the system are directly affected by the coupled reaction. Finally, we emphasize that the second law remains intact as, if the free energy change for the net process is determined (i.e., following the pathway of net flux), it will always be found to be exergonic.

## NESS in Enantioselective Catalysis

All chemical processes taking place spontaneously are trivially not at equilibrium: while a reaction is ongoing, there is net flux from starting material to product. Furthermore, catalytic processes are always cyclic reaction networks coupled to a spontaneous process, the conversion of substrates to products, and as such can be maintained at a NESS in which there is net flux around the cycle if their concentrations are chemostated. This observation does not appear particularly useful, as we are typically only interested in the products of the catalytic process. However, it is possible to construct reaction networks in which what we would normally identify as the substrate of the reaction takes the role of “catalyst” and one of the intermediates is the “product”.

### Enantioselective Synthesis by Harnessing a NESS

Using this perspective shift, it is possible to explain how, by harnessing a reaction network maintained at a NESS, we can convert a racemic or achiral starting material to a highly enantioenriched mixture in up to a 100% chemical yield. Several chemical strategies have been reported,^9^ as well as examples that take advantage of photochemical steps^22^ in the cycle. We shall demonstrate the general principles that underlie all such systems in the context of a minor enantiomer recycling (MER) reaction introduced by Moberg.^23^

**Figure 5:**
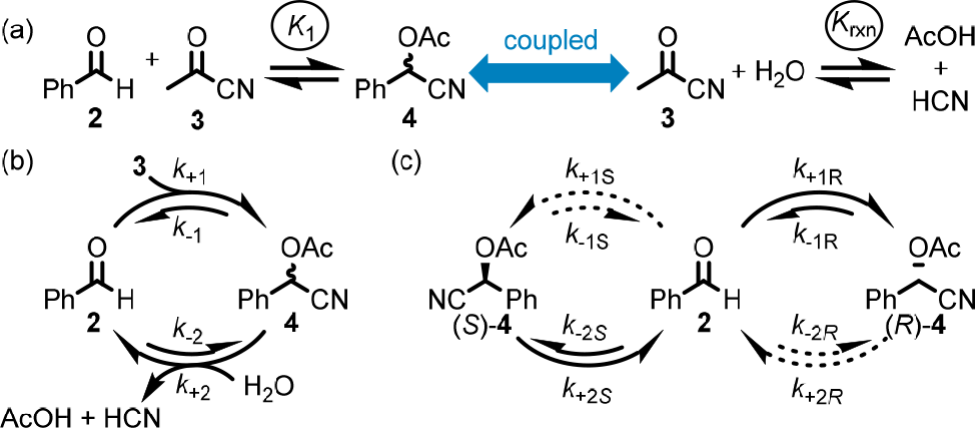
Moberg’s MER strategy. (a) The coupled reactions that form the reaction network. (b) The simple network formed when the reactions are coupled. (c) MER reaction network in which the rate constants for the enantiomeric pathways are distinct (**3**, H_2_O, AcOH, and HCN omitted for clarity).

In the MER approach, formation of an acylated cyanohydrin by reaction of an aldehyde with acylcyanide is coupled to the overall hydrolysis of the same acylcyanide reagent (Figure 5a). This is intuitively reasonable; formation of acylated cyanohydrin **4** by reaction of aldehyde **2** with acyl cyanide (**3**), followed by hydrolysis of **4** to regenerate **2** accomplishes the overall hydrolysis reaction (Figure 5b, cf. Figure 2), confirming that the aldehyde substrate mediates the coupled reaction. If the concentrations of **3**, H_2_O, AcOH, and HCN are chemostated to values inconsistent with *K*_rxn_, this coupling can potentially distort the steady-state concentrations [**2**] and [**4**] from the values predicted by *K*_1_ (i.e., a NESS is achieved; SI section 2.1). Practically, this is not useful, as product **4** will only accumulate if *k*_+1_ ≫ *k*_+2_, and so product formation is maximized when hydrolysis is minimized.

To see the power of this approach, we must recognize the following:

(i) **4** is chiral, and so the simple cycle (Figure 5b) can be rewritten as linked enantiomeric networks (Figure 5c).
(ii) Although introducing a catalyst does not alter the corresponding equilibrium constant, it can distort the relative proportions of species at NESS as these depend on the corresponding rate constants.
(iii) Enantioselective catalysts can potentially influence the two enantiomeric networks differently, allowing one enantiomer of **4** to dominate.

The authors demonstrated that introducing an enantioselective catalyst for the formation of (*R*)-**4** (*k*_+1*R*_ > *k*_+1*S*_) alongside an enzyme that stereoselectively hydrolyzes (*S*)-**4** (*k*_+2*R*_ < *k*_+2*S*_) under conditions in which both reactions take place simultaneously, gave (*R*)-**4** in higher enantiomeric excess than either catalyst alone. One way of thinking about the role of the two catalysts is that the hydrolysis catalyst corrects the “mistakes” of the acylation catalyst, which intuitively could lead to higher selectivity (SI section 2.4). Unlike classical approaches, in a well-optimized system, the final enantiopurity, *er*_final_, can approach the product of the enantiopurities, *er*_1_*er*_2_, obtained in the individual reactions when run under comparable irreversible conditions, and the yield can approach 100%.

Other aspects are less obvious. First, in addition to the requirements that the coupled reaction is chemostated to be spontaneous and that the system displays kinetic asymmetry, it is essential that the relative rates of different processes are properly arranged for the system to display both stereoselectivity and high yield—if the formation of **4** from **2** and **3** is highly stereoselective but slow and the hydrolysis reaction is highly stereoselective and fast, although at NESS **4** will be enantioenriched, aldehyde **2** will dominate. These requirements can be described quantitatively using very simple equations (SI section 2.2).

Second, qualitatively it should be obvious that the time taken to achieve the NESS is important—if the reaction must be run for a long period before it is achieved, during which time **3** is consumed continuously, the system is chemically extremely inefficient. Overall, the distribution of species within the network and the time taken to achieve steady state are functions of the reaction rates and equilibrium constants,^24^ as well as the steady-state concentrations of the species involved in the coupled reaction, all of which are potential points for optimization.

### Summary

We have focused on a single example, but the general features of such systems are common; if a catalytic reaction network is coupled to a chemostated spontaneous process, it is possible to generate a NESS in which the relative concentrations of species of interest are significantly perturbed. If this allows a desired species to be amplified, then this can be synthetically useful. This is of most obvious use in enantioselective synthesis, but this concept is of potential interest if a reaction network can give rise to any otherwise hard to access species.

## Information Ratchet Molecular Motors Operating Continuously at NESS

First we must differentiate molecular motors that operate continuously in a NESS, which are designated as “information ratchets”, from energy ratchets motors and switches operated in a stepwise manner.^10b^ In the former case, coupling a reaction network associated with molecular motion to a spontaneous chemical process can result in continuous net directional motion provided certain conditions are met.^13^ In the latter, external intervention is required to switch the system between different states. Importantly, although at the point of switching (e.g., change of pH, redox potential) the system is not at equilibrium and net flux between species will take place as it relaxes, once equilibrium is reached the system will remain at an equilibrium steady state until the next stimulus is applied.

### Information Motors—Escaping Detailed Balance

**Figure 6:**
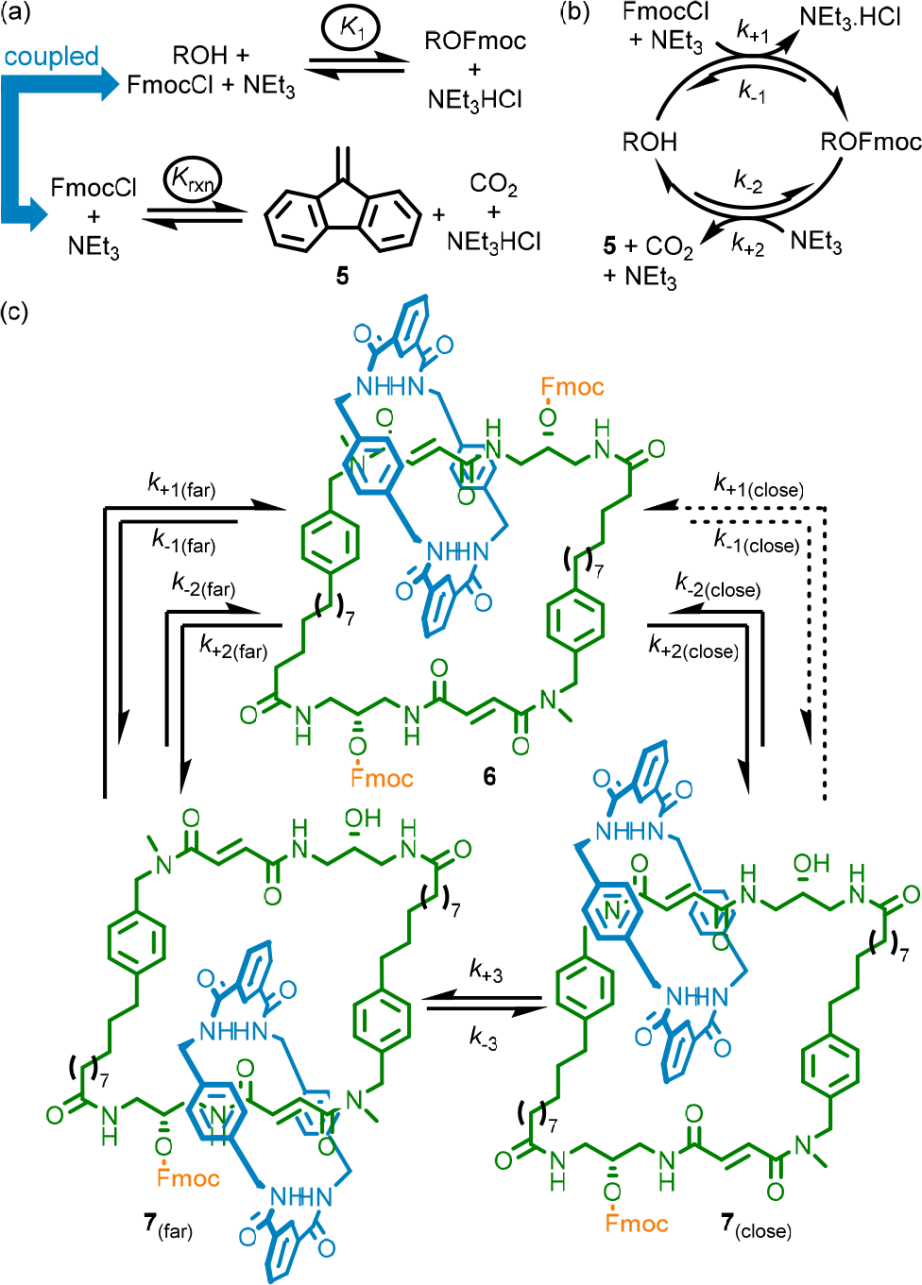
Information ratchet motor **6**. (a) The reactions that are coupled to generate directional motion in **6**. (b) The network that results from the coupling of these reactions. (c) Schematic representation of the operation of **6** (FmocCl, CO_2_, NEt_3_, and NEt_3_·HCl omitted for clarity).

For a molecular motor to operate continuously at NESS it should be obvious that detailed balance must be broken—the operation of a motor implies that there is net directional flux along a mechanical coordinate. For example, catenane **6**(25) operates continuously to generate net motion of the small blue ring around the larger green ring by carrying out the linking/unlinking of the two compartments via two chemically distinct pathways: base-mediated Fmoc ester cleavage and Fmoc-ester formation from FmocCl. This corresponds to the coupling of the reversible formation of the Fmoc ester, which acts as a gate to block mechanical motion, with the spontaneous conversion of FmocCl to dibenzofulvalene (**5**) and CO_2_ (Figure 6a).

The coupling of these reactions can distort the relative concentrations of an Fmoc carbonate and the corresponding alcohol even in a simple reaction network (Figure 6b). The final requirement to achieve continuous directed motion is kinetic asymmetry of the reaction cycle, corresponding to pirouetting of the two rings in catenane **6** (Figure 6c). This arises because the rate constants for the reaction with FmocCl differ in the two co-conformations of alcohol catenane **7**; Fmoc-ester formation proceeds faster for **7**_(far)_ than **7**_(close)_ (i.e., *k*_+2(close)_ < *k*_+2(far)_). This results in net clockwise movement (as drawn) of the smaller ring, even though the major pathway of Fmoc cleavage occurs to give **7**_(far)_ and **7**_(close)_ with equal probability (i.e., *k*_+1(close)_ = *k*_+1(far)_).

The operation of catenane **6** at NESS can be predicted mathematically—indeed the equation for the ratchetting constant (SI section 3.1) has the same form as that of eq 2 (Quantitative Descriptions of Simple Reaction Networks, Figure 4), despite the system appearing to be more complicated. The operation of **6** was demonstrated by carrying out the individual steps of the cycle separately on an isotopomer of **6** in which one of the fumaramide stations was deuterated to allow ^1^H NMR analysis. It was then demonstrated that both processes can proceed simultaneously to generate an autonomous motor.

### Ambiguity in the Analysis of “Autonomous” Switches and Motors

It was necessary to demonstrate the operation of catenane **6** indirectly because there is no single observable that can quantify the directed motion of a freely rotating motor. The same approach has been taken to demonstrate the behavior of all information-ratchet-based rotary motors reported to date.^26^ Indeed, compared with the operation of such networks in catalysis where product *ee* provides a clear observable, the autonomous operation of molecular motors can be opaque, even when the chemistry involved is simple.

**Figure 7:**
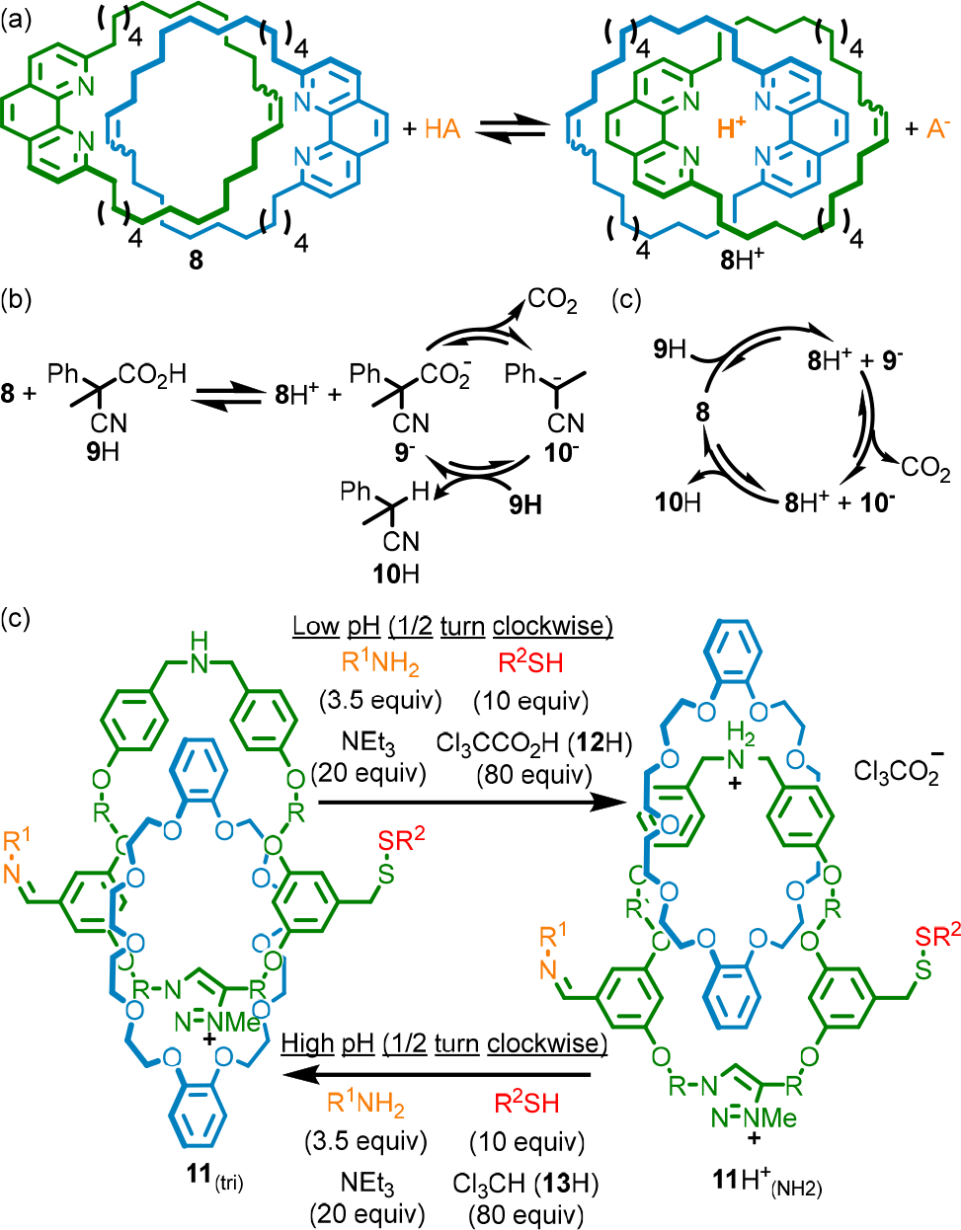
(a) Switching of catenane **8** on protonation. (b) A reaction network in which switching of **8** is not coupled to the conversion of **9**H to **10**H. c) A cyclic network in which the switching of **8** is coupled to the conversion of **9**H to **10**H. (c) Energy ratchet molecular motor **12** that completes a full turn either on addition of CCl_3_CO_2_H (**12**H) or by sequential addition of acid then base (R = (CH_2_)_3_, R^1^ = NH-3,5-di^t^Bu-benzoyl, R^2^ = 2,5-di-Me-benzyl).

For example, Di Stefano reported the operation of molecular switch **8** using carboxylic acid **9**H.^27a^ The dominant co-conformation of **8** under neutral conditions is that in which the phenanthroline units are far apart. Protonation of **8** gives rise to **8**H^+^ with concomitant relative rotation of the macrocycles to give a new dominant co-conformation (Figure 7a). Carboxylic acid **9**H, once deprotonated to give **9**^–^, undergoes spontaneous decarboxylation to give anion **10**^**–**^ which can then be reprotonated to give **10**H. Combining catenane **8** and acid **9**H gives rise to salt **8**H^**+**^·**9**^**–**^. Carboxylate anion **9**^**–**^ can then undergo decarboxylation to give a new salt **8**H^+^·**10**^**–**^. It is at this point that two different extreme pathways can be envisaged; **10**^**–**^ can be protonated by either another molecule of **9**H (Figure 7b) or by the same molecule of **8**H^+^ that initially accepted the proton (Figure 7c). Proton transfer is often mechanistically unimportant, but if one pathway is followed exclusively, it has consequences for how this system should be interpreted.

If **9**H is the proton donor, the switching process is not coupled to the decarboxylation reaction; the catenane simply remains protonated until the system runs out of **9**H (Figure 7b). If the system is chemostated to a steady state (i.e., [**9**H], [**10**H], and [CO_2_] are held constant), then there is no net flux between **8** and **8**H^+^. Conversely, if **8**H^+^ is the proton donor (Figure 7c), the reaction network is cyclic and there is net flux at steady state; the molecular switch moves back and forth between the co-conformational states, **8** and **8**H^+^, once for each decarboxylation event, and thus the switch could be said to be operating autonomously and continuously in the presence of excess **9**H. In fact, both pathways may contribute, and their relative importance may depend on the reaction conditions.^27b^

Similarly, Cl_3_CCO_2_H (**12**H), which undergoes a base-catalyzed decarboxylation to give CHCl_3_ (**13**H), has been used to operate catenane (e.g., **11**) and rotaxane molecular motors.^28^ Although the motion superficially appears to be coupled to the conversion of **12**H to **13**H, we must analyze the system to understand how its behavior should be interpreted. In this case, the conclusion is unambiguous: because addition of **12**H results in a single cycle of operation—the ring first moves clockwise from the triazolium station past the acid-labile hydrazone “gate” to the protonated ammonium station, and then when the pH rises it returns to the triazolium station past the base-labile disulfide “gate”—catenane **11** operates by an energy ratchet mechanism.

Thus, the mechanical motion in **11** is not formally coupled to the conversion of **12**H to **13**H. Unlike in the case of information ratchet **6**, the maximum work that motor **11** can perform per cycle is not a function of the overall free energy change associated with this process (SI section 4); the maximum work that can be performed at each stage of the cycle is simply determined by the energetic difference between the two co-conformations, as it is if **11** is operated by simple addition of acid followed by base.^29^ Indeed, the operation of switches and energy ratchet motors such as **11** using reagents that change the properties of the system (e.g., pH) in a time-dependent manner but whose conversion is not coupled to the network is conceptually identical to the simple addition of one reagent (e.g., HCl) followed by a counteracting other reagent (e.g., NEt_3_).^30^

### Summary

Although coupling a spontaneous process to a reaction network to generate an NESS has a very different objective in the case of synthesis (distort concentrations of key species) than in the operation of molecular motors (escape detailed balance), the principles are identical. Information motors have been shown to generate a continuous net directional motion if the coupled reaction is spontaneous. These elegant examples of synthetic molecular motors can provide a tractable platform to explore concepts related to biological molecular motors, such as ATP-synthase,^31^ which operate according to the same principles. However, even in relatively simple systems, ambiguity can arise; the details of the reaction network and the associated mechanism and kinetics are important. Finally, although a cyclic reaction network coupled to the spontaneous reaction is required to drive continuous repetitive motion, the motion itself need not be cyclic. For example, Leigh has demonstrated a prototypical system that could operate as a linear motor but whose behavior also relies on a cyclic reaction network,^32^ as do the operation of linear biological motors such as kinesin.^33^

## Supramolecular Assembly at NESS

The assembly of small molecules to generate supramolecular polymers takes place reversibly to give a thermodynamically favorable assembly. This general principle has been used to create a wide range of structures based on various non-covalent interactions, including H-bonding, π–π interactions, metal–ligand interactions, and host–guest chemistry, for a range of proposed applications.^34^ In all cases, an equilibrium steady state is achieved, in which there is no net flux between the molecules in solution and the aggregate. As with simple molecular switches, it is possible to switch supramolecular polymers between different states by varying the conditions or applying other stimuli.^34,35^ Once again, although at the point of switching the system is away from equilibrium, over time a new equilibrium state is reached.

As with artificial molecular motors,^10^ there has been a surge of interest in supramolecular materials whose assembly is influenced by an ongoing chemical reaction with the ultimate aim of generating self-assembled structures in a NESS.^11,12^ However, unlike motors, where directed molecular motion is correlated with flux through the reaction network, requiring the system to escape detailed balance, the consequences and benefits of a supramolecular material maintained at a NESS are less obvious.

We will initially focus on the assembly of actin monomers coupled to the hydrolysis of ATP,^36^ an important biological process that clearly meets the criteria and is often cited as inspiration for the development of artificial systems. However, misunderstandings of how self-assembly takes place in the case of ATP-actin are common. After this brief but necessary detour, we will return to synthetic systems.

### A Simplified Description of Actin Self-Assembly

The process of actin assembly is complicated *in vivo*, where proteins bind to the filament and influence its behavior.^36^ For simplicity, we will focus our discussion on the *in vitro* process that takes place in the presence of ATP.^37^ To simplify the discussion further, we shall initially focus on the process of adding a single monomer unit to a preformed ATP-actin filament. The discussion that follows is based in part on an excellent review^36b^ and detailed discussion of actin polymerization kinetics reported by Pollard.^38^

**Figure 8:**
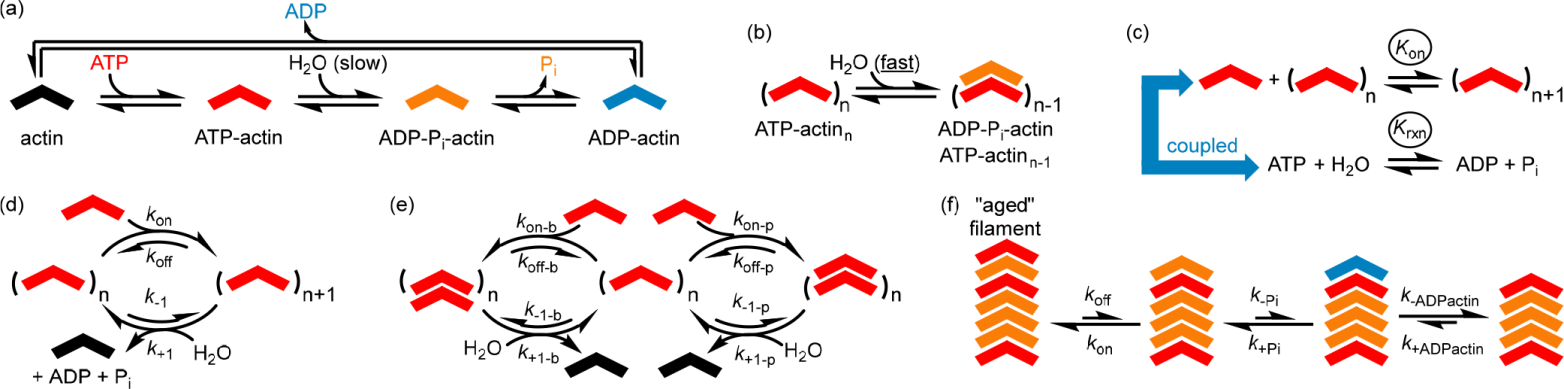
Simplified models of actin polymerization at NESS. (a) The behavior of actin in solution with ATP; ATP-actin hydrolyzes slowly. (b) ATP-actin hydrolyzes rapidly when assembled in an actin filament. (c) The coupled processes involved in the assembly of ATP-actin to a preformed filament. (d) A simple reaction network that confirms the coupling of assembly and hydrolysis even when the details of the hydrolysis process are ignored. (e) An expanded model of ATP-actin self-assembly that can be used to account for treadmilling. (f) A schematic that demonstrates that the disassembly of aged actin filaments is qualitatively different from the disassembly of a simple supramolecular polymer; ADP-P_i_-actin monomer units in the bulk are kinetically stable and strongly bound to the filament but can rapidly lose P_i_ at the termini, resulting in the dissociation of ADP-actin.

In the presence of ATP,^37^ actin is in equilibrium with an ATP-actin complex, which is strongly favored (Figure 8a). This undergoes a very slow hydrolysis to generate a new ADP-P_i_-actin complex. Subsequent dissociation of P_i_ and ADP regenerates actin. This process is slow and will henceforth be ignored for simplicity.^39^ Conversely, when an ATP-actin monomer associates with an actin filament, which is also a favorable equilibrium, the hydrolysis process is significantly accelerated (Figure 8b). From this very simple description, it is obvious that the hydrolysis of ATP is coupled to the reversible addition of an ATP-monomer unit to the filament (Figure 8c), and we can draw a simple reaction network to demonstrate the cycle (Figure 8d).

The following observations confirm that the addition of an ATP-actin monomer to the ATP-actin filament coupled to ATP hydrolysis can achieve a NESS:

(i) ATP hydrolysis is spontaneous under the conditions of assembly (coupled reaction).
(ii) The system displays kinetic asymmetry.
(iii) The reaction network includes both the assembled and disassembled states.

A slightly more detailed description is needed to account for one of the most well-known features of actin filament behavior, “treadmilling”. Under certain conditions, monomer units are added to one end of a filament and lost from the other end without any overall change in length, resulting in the filament appearing to move across a surface.^36,38^ We start by noting that the two ends of the filament are inequivalent due to the nonplanar ATP-actin monomer. Thus, new monomer units can be added to either the “barbed” or “pointed” end of the filament (Figure 8e). The distinguishable ends could lead to the simple but incorrect conclusion that ATP-actin monomers bind to the two ends with different association constants. However, as identified by Pollard,^38^ adding the ATP-actin monomer to either end of the filament results in indistinguishable products—(ATP-actin)_*n*_ → (ATP-actin)_*n*+1_ in both cases—and so the association constants are thermodynamically required to be the same.

However, the association constant for the addition of monomer units at the barbed end has been measured to be more favorable.^38^ This contradiction can be rationalized qualitatively by noting that the behavior of a system in a NESS is controlled by kinetic considerations: although the association constants (i.e., the ratio of on/off rate constants) at the barbed and pointed ends must be the same, the rate constants themselves can differ. Similarly, the overall rate of ATP-actin hydrolysis and monomer dissociation (*k*_+1_) at the barbed and pointed ends need not be identical. Indeed, the hydrolysis process itself is actually quite complicated (see below). The conclusion is that, regardless of the finer details, the *apparent* difference in experimentally determined association constants for ATP-actin to the pointed and barbed ends of an actin filament arises due to kinetic differences in processes taking place.

The other widely noted feature of actin filaments is their unusual disassembly kinetics when compared with those of simple supramolecular polymers. The rate constants of association (*k*_on_) and dissociation (*k*_off_) of a simple supramolecular polymer are directly linked by the corresponding equilibrium constant, but disassembly of aged actin filaments can occur more rapidly than would be predicted by consideration of *k*_off_ of the ATP-actin monomer unit when the system is deprived of ATP. To qualitatively explain this observation, we must depart from our simple model in which the bulk filament is composed solely of ATP-actin and note that ATP-actin hydrolysis is thought to take place with equal rate throughout the filament.^38^ Thus, an actin filament is actually composed of a random arrangement of ATP-actin and hydrolyzed monomer units (Figure 8e), the exact proportion of which will depend on the “age” of the filament—the probability of a monomer unit being hydrolyzed depends on how long it has been embedded in the filament.

This much is widely accepted, but it is sometimes suggested that the observed rapid disassembly kinetics of aged filaments results from “high-energy” ADP-actin being trapped in the bulk of the filament. First, ATP-actin hydrolysis does not lead directly to ADP-actin; the initial hydrolysis product is ADP-P_i_-actin, whose measured association constant to the filament is very similar to that of ATP-actin—the hydrolysis of ATP does not significantly alter the binding of the monomer unit to the filament.^38^ Second, P_i_ dissociation is an extremely slow process (*t*_1/2_ ≈ 6 min), so ADP-P_i_-actin units in the bulk of the filament are kinetically long-lived polymerization intermediates.^38^ Third, the eventual dissociation of P_i_ produces an ADP-actin unit embedded in the filament, but it should be noted that ADP-actin itself assembles into filaments, albeit with a lower critical concentration—this is not an unstable arrangement.

Taking these points into account, the rich depolymerization behavior of actin filaments arises from its dependency not only on the monomer (ATP-actin) concentration, as for a simple supramolecular polymer, but also on the age of the filament (degree of hydrolysis), the concentration of P_i_, and the different rates of dissociation of P_i_ and ADP-actin at the barbed and pointed ends. Thus, once depolymerization starts, it can proceed extremely rapidly as the ADP-P_i_-actin or ADP-actin units, which dissociate more rapidly than ATP-actin,^38^ reach the filament ends. On the other hand, while the filament is growing, the polymer remains remarkably resistant to degradation, despite the hydrolysis of internal monomer units.

To conclude, the assembly of actin filaments clearly produces a self-assembled supramolecular material that can be formed in a NESS with unusual and interesting properties. However, it is hard to draw strong, general conclusions about the potential properties and applications of synthetic systems based on this example. For instance, it is not automatic that treadmilling or similar processes will occur in all such materials. The rapid breakdown of disassembling actin filaments might be more general, but we must re-emphasize that this is not because of the trapping of “high-energy” monomer units within the structure. Similarly, there is no simple link between the free energy of ATP hydrolysis and any free energy “stored” in the self-assembly (see below). Moreover, the *in vivo* behavior of actin is far more complicated than our simple model: proteins regulate every aspect of the process, including nucleation, elongation, severing, and even cross-linking filaments.^36^

### Ambiguity in the Analysis of Artificial Supramolecular Materials Proposed to Achieve a NESS

**Figure 9:**
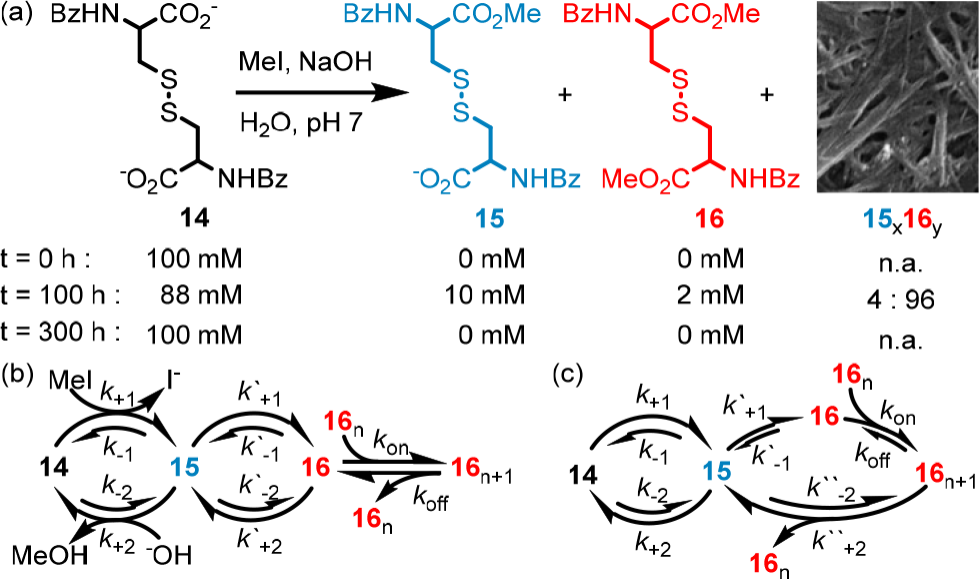
(a) Transient assembly of a gel by the conversion of **14** to **15** and **16**. The results can be explained by two extreme reaction networks in which (b) assembly is not coupled to the hydrolysis of MeI (hydrolysis of **16** only in solution) or (c) it is strongly coupled (hydrolysis of **16** only in the gel state); the real network is likely to be intermediate between these extremes. Rate constants *k*_±1_ and *k*_±2_ refer to reaction of MeI to generate an ester and hydrolysis, respectively. MeI, I^–^, ^–^OH, and MeOH are omitted from the majority of the network for clarity.

Returning to artificial systems, many examples have been described in which, broadly speaking, a species that does not self-assemble reacts to give a monomer unit that does.^11^ A subsequent reaction then destroys the monomer units to regenerate the dispersed starting material. In one example to stand for all, van Esch reported one of the earliest examples of such systems,^40^ which is based on an ester supramolecular gelator system that relies on the presence of MeI (Figure 9a). Dicarboxylate **14** is freely soluble under the reaction conditions, but in the presence of MeI, **14** is converted first to monoester **15** and then to diester **16**, which spontaneously self-assembles into a gel structure. Hydrolysis of **16**, first to **15** and then to **14**, returns the system to its original, fluid state.

The experiment described clearly generates a transient self-assembled state (the gel) in the presence of MeI that is lost once MeI is depleted. The formation and destruction of monoester **15** and diester **16** are also clearly coupled to the alkaline hydrolysis of MeI (cf. Quantitative Descriptions of Simple Reaction Networks). However, it is not obvious that the self-assembled material is directly affected by the coupled reaction; the same gross behavior would be observed if the gel fibers were in equilibrium with ester monomer unit **16** but ester formation/hydrolysis took place exclusively in solution (Figure 9b) or if the hydrolysis of **16** took place exclusively in the fibers (Figure 9c). In the former case, the relative concentrations of gel and monomer **16** are unaffected by the coupled reaction, and thus, although a NESS can be established in which the concentrations of **15** and **16** relative to **14** are distorted, the gel is “at equilibrium” with monomer **16** at any given instant and its properties are essentially identical to the structure achieved by simply mixing the same proportions of **14**, **15**, and **16**.^41^ In the latter case, the self-assembled state is directly influenced by the coupled reaction, as in ATP-actin polymerization. Prins suggested that systems corresponding to the former situation be described as forming under “dissipative conditions” to differentiate them from those in which self-assembly is directly coupled to a driving reaction, which they term “dissipative self-assembly” or “driven self-assembly”, depending on whether kinetic asymmetry is present.^11b,12a^

This is not to say that systems have not been reported that show behavior strongly suggestive of being able to achieve a NESS. van Esch later described the gelation of monoester **15** formed by reaction of **14** with SO_4_Me_2_.^42^ Under these conditions, the gel displayed nonlinear dissolution kinetics of gel fibers and their simultaneous growth and shrinkage under some regimes. Similarly enticingly, Hermans observed oscillation in the self-assembly of functionalized perylene diimides when simultaneous reduction and oxidation of monomer units took place,^43^ and Fletcher observed chemical oscillations under conditions where a thiol was converted into a disulfide amphiphile and subsequently to a non-assembling disulfide.^44^

However, as noted by Prins,^12a^ although the properties of the gel reported by van Esch formed in the presence of SO_4_Me_2_^42^ are reminiscent of the behavior of supramolecular biopolymers,^36,45^ only detailed kinetic analysis of reactions in the dispersed and aggregated states would unequivocally confirm the form of the reaction network and that the required kinetic asymmetry is present to achieve a chemically driven NESS.^13^ Obtaining these data under heterogeneous conditions is extremely challenging and complicated by confounding factors. In van Esch’s system, gel formation will affect the mass transport of reagents, and the system is unstirred.^42^ Conversely, Hermans’s system^43^ requires stirring for the observation of chemical oscillations, which the authors note brings mechanical fragmentation of the fibers into play.^46^ Fletcher’s system is even more complicated, as it requires stirring to ensure mixing between bulk fluid phases, and the aggregated micellular state is also subject to sheer forces.^44^ Given that these other processes also provide a path for free energy dissipation in addition to the ongoing coupled reaction, a complete treatment of these networks would require these factors to be considered explicitly.

### Summary

Based on the above, it is hard to escape the conclusion that the assembly of artificial supramolecular materials that can in theory achieve a chemically driven NESS with respect to their monomers has yet to be unambiguously demonstrated by confirming the full form of the network and the presence of kinetic asymmetry. That is not to say that the systems reported are not elegant or exciting—we have highlighted several with intriguing properties—but simply that it is very hard to unambiguously demonstrate that the self-assembly process is coupled to the driving reaction and if kinetic asymmetry is present because the required data are hard to obtain. Furthermore, there is no simple observable (e.g., directed motion, enantioselectivity) that would unequivocally confirm the nature of the network, which leads to a reliance on phenomenological observations. In the case of the systems highlighted, these observations are very enticing. However, we note that transience (e.g., Figure 9), which is commonly reported, is not sufficient to confirm that a NESS involving the supramolecular assembly is achieved or possible.

## An Appeal for Clear Language and Notation

As highlighted in the Introduction, the language used to discuss chemical systems in a NESS is still in development. Thus, some widely used terms may mean different things to different people.^9−11^ Other terminology, while evocative, can be actively misleading, particularly when it draws apparent equivalence between macroscopic and molecular-scale processes. Here we address key examples and related issues. As scientists, we should strive to define concepts as clearly and accurately as possible; otherwise, our scientific discourse will be confused and incoherent. Others have offered different definitions of some of the terms below, and the interested reader is directed to their writings.^10b,10d,10e,11a−11c,12^

### “Catalysis”

It is sometimes said that the molecular motor or the monomer of a self-assembled material “catalyzes” the coupled reaction. Although it is true that these species mediate the reaction without being consumed, this does not necessarily mean that they correspond to what most chemists would consider a catalyst.^47^ Chemists usually understand “catalysis” to mean that the overall energy barrier of a transformation has been lowered relative to that of the uncatalyzed pathway. In some cases (e.g., catenane **6**), the rate of the coupled reaction is higher outside of the reaction network. Thus, we prefer “mediates” to “catalyzes”, as it covers all eventualities. That said, “catalyze” is obviously correct in analogous biological systems, which are highly optimized.^31^

### “Reversible” vs “Irreversible”

Throughout we have treated all reactions as fully reversible. This is necessary because all reactions are technically reversible, with the forward and reverse rate constants related by the corresponding equilibrium constant. Thus, it is never accurate to set a reverse rate constant arbitrarily to 0—this would correspond to the reaction free energy being infinite and negative. However, it is true that a reaction can be practically irreversible if one product is lost from the system. For example, in the case of catenane **6**, the evaporative loss of CO_2_ during the operation prevents the cleavage reaction from reversing at an appreciable rate. This apparent problem can be resolved by setting [CO_2_] to a low but realistic value once an equation for *r*_0_ has been constructed (SI section 3.1). More generally, it is obviously appropriate to take mathematical limits once equations describing a network have been constructed. However, arbitrarily ignoring processes *en route* to these equations can lead to physically inconsistent results.

### Arrow Notation

**Figure 10:**
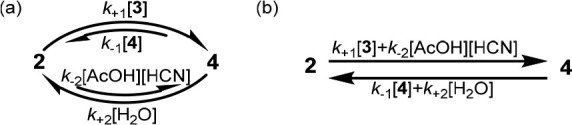
Comparison between (a) the standard representation of the reaction network in Figure 5b making use of equilibrium arrows (**⇌**) and (b) the equivalent network in which simple arrows (⇄) represent composite processes.

The confusion over irreversibility can be exacerbated by confusion over the meaning of different arrows. We suggest that equilibrium arrows (**⇌**) should be reserved for a reversible process obeying microscopic reversibility (*K*_eq_ = , e.g., Figure 10a). Simple arrows (⇄) should be used to indicate that the processes are not bound by microscopic reversibility. These are appropriate when indicating a photochemical exchange (see below) or if the arrows represent a composite process; it can sometimes be useful to combine multiple pathways linking species over a single arrow (Figure 10b) to simplify the depiction of complex systems.

### “Transient”

The word “transient” means that a property of a system persists for a defined period. Any ongoing spontaneous process may result in a transient change (e.g., a reaction intermediate is colored) that persists until the reaction is complete (e.g., the intermediate is consumed). It is often used in the context of supramolecular materials whose formation is associated with an ongoing chemical reaction: the material forms and then disassembles once the reaction process is complete. “Transient” thus has a real physical meaning as an observable property of a system. However, transience is not in itself evidence of a NESS.

### “Out of Equilibrium”

It is often said in the context of both molecular motors and self-assembled materials that a system is “out of equilibrium”—indeed, living systems, which are cited as inspiration for many artificial systems, are often described as operating “far from equilibrium”. This term is also often applied to describe the molecular motor or self-assembled structures themselves. The latter is problematic because it is not clear what it means for a molecule or self-assembled structure to be out of equilibrium in the abstract sense.^48^ More correctly, the relative concentrations of molecules can be out of equilibrium (i.e., not according to the corresponding equilibrium constants) with respect to those of species with which they can potentially exchange, but this is seldom specified in such discussions. We also note that any system prepared in a metastable system would meet this definition; NaOAc-based hand warmers are useful, but no one suggests they are cutting-edge science.

Returning to the whole system, if a spontaneous process is taking place (e.g., MeI hydrolysis) the system is clearly not at equilibrium, and so in this sense the term “out of equilibrium” is appropriate. However, this is true of any system that is undergoing a spontaneous change—a sodium metal in water is out of equilibrium, as is, less excitingly, an ester in alkaline solution. Thus, it is important to specify *how* the spontaneous process is affecting the properties of the system to decide if it is non-trivially out of equilibrium, by which we mean that a NESS can be achieved. Importantly, this also requires us to identify which parts of the system are affected by the coupled reaction. As shown in Quantitative Descriptions of Simple Reaction Networks, reactions not directly coupled to the spontaneous process, or part of a cycle in which at least one step is, are not directly affected. Thus, simply saying that a system is out of equilibrium does not provide useful information about how the spontaneous process is coupled to the reaction network.

### “Dissipative”

The term “dissipative” is similar to “out of equilibrium”; indeed they are almost synonyms. Any process that takes place spontaneously is, by definition, out of equilibrium and will dissipate free energy (Δ*G* for the process is negative). If the system is chemostated such that the coupled reaction never reaches equilibrium, this dissipation will be continuous. However, as noted above, this is interesting only if the spontaneous process is coupled to the process of interest. The word “dissipative” also brings the focus toward the release of free energy, which can be problematic, as we will discuss next in the context of the word “fuel”.

### Semantic Objections to “Fuel”

We have avoided the words “fuel” and “waste” although they are routinely used to describe the substrate and product of coupled reactions (e.g., MeI and MeOH), leading to expressions such as “chemically fueled self-assembly” and “chemical fuel driven motor”. However, “fuel” is evocative, recalling macroscopic engines, and thus leads ineluctably to related images of a “fuel” molecule providing a “kick” that, for example, in the case of motors drives mechanical motion, which is physically incorrect. If one molecule undergoes a spontaneous reaction with another, they remain at thermal equilibrium with their environment—bonds are not “excited”, and molecules are not “pushed”. Indeed, because molecules exist in a low Reynold’s number regime, momentum is meaningless in the discussion of molecular motors.^13b^ As noted by Astumian, there is no “judo throw” ^13f^ that directly transfers energy from the “fuel” molecule to the system being discussed.

Related to this is a tendency to suggest that some fraction of the free energy of a chemical change associated with a “high-energy fuel” can be used to do work against an opposing force in molecular motors in a specific step of the cycle. However, Hill demonstrated that it is not possible or meaningful to attempt to identify in which step of the reaction cycle chemical potential is transduced into mechanical energy in the operation of a molecular motor.^49^ Similarly, in the context of supramolecular materials, it is often suggested that the reaction between the “fuel” and a non-self-assembling monomer unit (e.g., **14**, Figure 9) produces an “activated” monomer (**16**) that then self-assembles. This process is sometimes drawn as energetically uphill to signify “activation”, despite spontaneous chemical reactions being exergonic. This is connected to the idea of some proportion of the energy associated with a “high-energy fuel” being stored in the self-assembled structure.

These ideas perhaps stem from the language that biochemists and biologists use when discussing and thinking about the energetics of bond-breaking and -making:^50^ it is sometimes said that “high-energy phosphate” is produced during ATP hydrolysis or that the breaking of a “high-energy phosphate bond” in ATP is used to “fuel” biological processes. Chemists should recognize the fallacy of such claims: although the exchange of a weaker bond for a stronger one (e.g., C–I for C–OH in MeI hydrolysis) is strongly indicative of an exothermic process, it is the formation of a strong bond that results in the release of free energy. The loss of a weak bond simply minimizes the energetic cost of doing so.

### Technical Objections to “Fuel”

If we examine the equations that describe the rotation of catenane motor **6** and the work it can perform (SI sections 3.2 and 3.3), we arrive at more technical objections to the word “fuel”. These equations have the same features as all information motors operating at NESS, allowing us to reach general conclusions that contradict the macroscopic preconceptions the word “fuel” conjures:

(i) The motor can turn in either direction, regardless of the value of Δ*G*_rxn_, including if Δ*G*_rxn_ = 0, depending on the concentrations of the species in the coupled process.
(ii) The maximum work (Δ*w*_max_) of a “perfect” motor can exceed Δ*G*_rxn_, as it also depends on the concentrations of the species involved in the coupled process.

Neither is consistent with a focus on the chemical properties of a fuel. The same coupled reaction can be used to drive the motor in either direction, and the same value of Δ*w*_max_ can be obtained if Δ*G*_rxn_ is small but the reaction components are chemostated far from equilibrium or if Δ*G*_rxn_ is large but the components are chemostated near to equilibrium. Instead, the continuous directed motion of a molecular motor such as **6**, and the maintenance of a NESS more generally, is driven by the free energy change associated with mass action, the flow of substrate to product, rather than some simple energetic property of the “fuel”.^13g^

Finally, the behavior of molecular motors made to work against an opposing force that exceeds their stall force can be inconsistent with our macroscopic expectations—forcing ATP-synthase to move backward reverses the direction of the coupled reaction; ADP and P_i_ are converted to ATP.^31^ Although a detailed discussion of the features ATP-synthase that lead to this behavior lies beyond this Perspective,^51^ it is not consistent with our experience of “fueled” motion in the macroscopic world—a car does not consume CO_2_ and produce gasoline if it is pushed backward, which would be the macroscopic equivalent.

### If “Fuel” Must Be Used

Given the above, we would obviously counsel against the use of “fuel” and “waste” and terms such as “chemically fueled”. We suggest that “substrate” and “product” and “chemically driven”, respectively, are more useful. Unfortunately, “fuel” is so widely used that it is hard to see it being readily supplanted.

Thus, it is important that at the absolute minimum its meaning is clarified so that it is useful. Although others have suggested definitions,^10b,10d,10e,11a−11c,12^ a key feature that is omitted is the need to define how the driving reaction is coupled to the process of interest. This leads to “fueled” being applied^10d^ equally to the operation of an information motor (driving reaction coupled to the directed motion, maximum work equal to the free energy associated with mass action, NESS can be achieved) and the operation of an energy ratchet motor (driving reaction not coupled to directed motion, maximum work not a direct function of the free energy associated with mass action, NESS cannot be achieved).

We propose that, whether a process is described as “chemically driven” or “fueled”, both the coupled reaction and the form of the reaction network that confirms it is coupled to the process of interest (directed motion, self-assembly) need to be clearly defined and demonstrated through appropriate kinetic measurements. In the cyclic network (Quantitative Descriptions of Simple Reaction Networks, Figure 4), conformational exchange between RCO_2_Me and R′CO_2_Me could be described as “chemically driven” or “fueled” by the hydrolysis of MeI to MeOH, whereas in the linear network (Figure 3), in which the conformational exchange process is not coupled to the hydrolysis of MeI, this would not be appropriate. Similarly, “fueled molecular motor” would be reserved for systems that operate via an information ratchet process, and “fueled self-assembly” would be reserved for systems in which the aggregated state is demonstrated to be part of the reaction network that consumes the “fuel” and kinetic asymmetry is present.

This does not mean that we rescind the objections above. Energy is still not transferred between molecules, and there is still no special energetic property of the “fuel” molecule that controls the system.

### Coupled Reaction Free Energy and Energy “Stored” in Self-Assembled Structures

Finally, the focus on molecules as “fuel” has also led to the idea of energy from the “fuel” being stored in self-assembled structures at NESS. Although a chemically driven reaction network can result in a thermodynamically unstable distribution between monomers and the self-assembled state that would spontaneously disassemble were the coupled reaction “switched off” (i.e., if all reaction components were removed), we hope it is now clear that the distribution obtained is a consequence of kinetic asymmetry, as well as the free energy change associated with mass action, and is not a simple function of the energetics of a “fuel”-to-“waste” reaction. Indeed, the same chemical potential could be stored in a NESS where a thermodynamically unstable aggregate is produced, often the focus of such discussions, such as one in which a stable aggregate was overpopulated.

### Summary

The above discussion is not intended to be needlessly pedantic. However, when molecular switches and energy ratchets are described as “out of equilibrium”, or similarly, when this term is applied to supramolecular polymers that are not part of the reaction network that includes the coupled reaction, it causes confusion. This can in turn obscure what has been achieved in an experiment and, in the worst cases, lead other researchers down blind alleys or prevent genuine advances from being properly identified. Similarly, if every system in which a reaction is taking place is thought to be “fueled” and some energetic property of the “fuel” is thought to be key to the process, this incorrectly draws focus onto the “fuel” molecule rather than the properties of the network (i.e., kinetic constants etc.) which govern the system and require more attention, in our view, than they are currently receiving. For example, in the case of catenane motor **6**, a focus on the properties of the “fuel” could tempt one to include the free energy change associated with the evaporation of CO_2_ in the maximum work that could be done. However, this step lies outside of the reaction network and so does not directly affect the behavior of the motor other than by limiting [CO_2_].

## Why Are Photochemical Processes “Special”?

**Figure 11:**
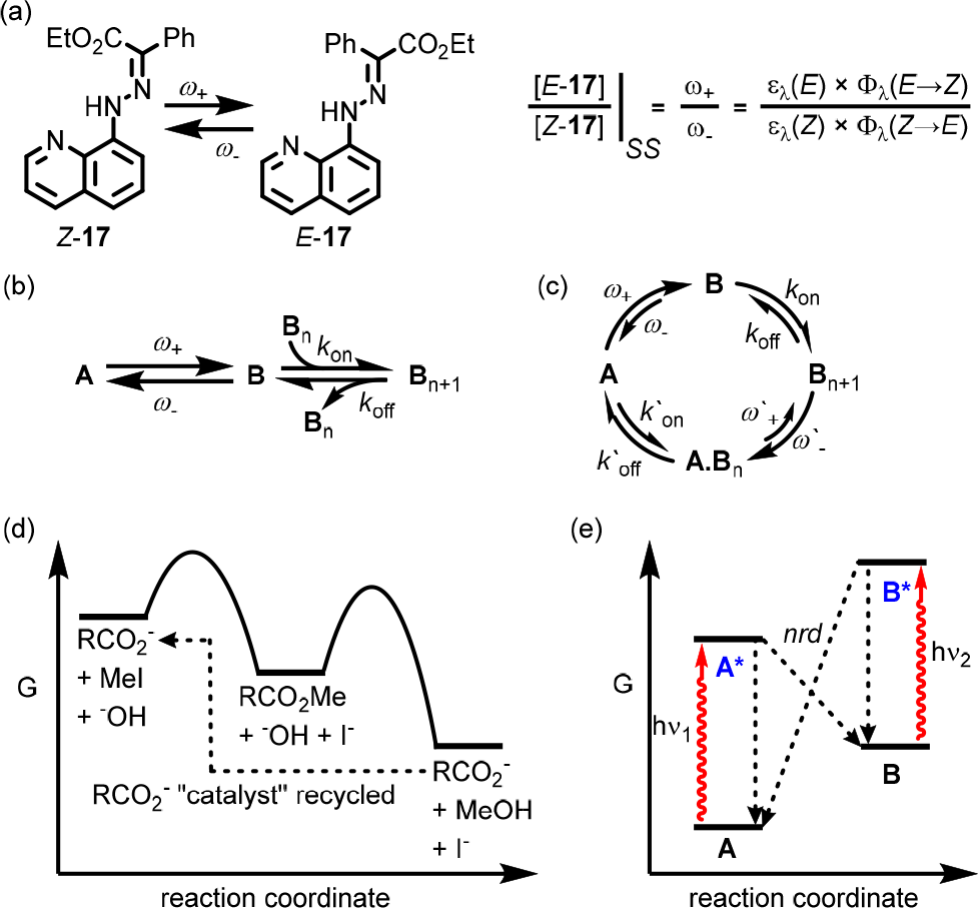
(a) Isomerization of hydrazone **17** and the associated equation for the *E*:*Z* ratio at PSS (ε_λ_(*E*/*Z*) = extinction coefficient at wavelength of irradiation λ; ϕ_λ_ = quantum yield of indicated isomerization process at wavelength λ). (b) “Linear” network for the assembly of monomer **B** under continuous irradiation in which the supramolecular polymer is at equilibrium with the dispersed monomer. (c) Cyclic network for the assembly of monomer **B** under continuous irradiation in which the supramolecular polymer is at a NESS (primed kinetic constants refer to processes involving the aggregated state). (d) Schematic potential energy diagram for ester exchange reaction (Quantitative Descriptions of Simple Reaction Networks) 1. highlighting that the overall cycle is thermodynamically downhill and takes place on a single surface. (e) Schematic potential energy diagram for the isomerization of **17** highlighting that the conversion of *Z*-**17** to *E*-**17** is thermodynamically uphill and that the process takes place on two different surfaces (nrd = non-radiative decay).

Put simply, they do not have to obey microscopic reversibility.^17,52^ This has allowed the development of elegant molecular motors by Feringa^53^ and others.^54^ To take a simple example, in the *E*/*Z* isomerization reaction of hydrazone photoswitch **17** (Figure 11a),^55^ ignoring any thermal isomerization processes,^56^ the apparent rate constant for conversion of *E*-**17** to *E*-**17**, ω_+_, upon irradiation with light of wavelength λ, depends on the extinction coefficient (ε_λ_) of *Z*-**17**, the intensity of the incident light (*I*), and the quantum yield of isomerization (Φ_λ_).^57^ A similar argument applies to the conversion of *E*-**17** to *Z*-**17**. Given that the values of ω_+_ and ω_–_ are not intrinsically related to the relative thermodynamic stability of *Z*-**17** and *E*-**17**, there is no reason for their ratio at the photostationary state (PSS) to be in accord with the corresponding equilibrium constant. This means that all^58^ photochemical exchange processes under continuous illumination automatically correspond to a NESS. Based on this, the key question to ask of a network that includes a photochemical step is whether it is coupled to the process of interest—the same question that we advocate should be asked of all chemically driven systems.

For example, if molecule **A**, which is fully dispersed in solution, is photoisomerized to give **B**, which forms a supramolecular aggregate **B**_*n*_, to determine whether the self-assembly is at a NESS under continuous irradiation^59^ we must determine if the network is “linear” or cyclic and if it displays kinetic asymmetry (cf. Quantitative Descriptions of Simple Reaction Networks). If the network is linear (photoisomerization only in solution; Figure 11b), although the relative concentrations of **A** and **B** at PSS do not accord with their relative thermodynamic stability, the relative concentrations of **B** and **B**_*n*_ obey the corresponding equilibrium constant. Conversely, if the network is cyclic (isomerization takes place both in solution and in the aggregated state; Figure 11c), the relative concentrations of **A**, **B**, and **B**_*n*_ may be perturbed. Furthermore, the isomerization of **B** to give **A** in the aggregated state could give rise to the non-aggregating monomers being incorporated into the assembly, even if this is not thermodynamically favorable.

Finally, we highlight that, whereas chemically driven processes take place on a single, continuous potential energy surface, photochemical processes take place on at least two surfaces, the ground and excited states.^60^ Thus, photochemical processes begin with a large “uphill” step (Figure 11e) that corresponds to a proportion of the absorbed photon’s energy being transferred to a molecule to generate an excited state. Decay to the ground state of an isomer of the starting material with higher chemical energy can then take place. It seems reasonable to describe this isomer as “activated” (i.e., metastable) relative to the starting material, and it is possible to quantify the amount of energy it “stores” from the incident photon. If subsequent steps that re-isomerize the molecule to its initial state are coupled to the self-assembly of the metastable isomer, it is easy to trace the absorbed photon’s energy to where it is “stored” by following the single molecule.

Conversely, chemically driven processes are “downhill” over the cycle (Figure 11d),^61^ and there is no moment where energy is “added” to a specific molecule. For example, RCO_2_Me, produced by reaction of RCO_2_^–^ with MeI, contains atoms from both starting materials—Which atoms are “activated” and which are “deactivated”? Despite this, it is sometimes said that a monomer unit becomes “activated” by reaction with a “fuel” molecule to drive self-assembly, alongside energy diagrams in which the reaction of the monomer with the “fuel” is shown as energetically uphill. Both may be inspired by drawing a false equivalence with photochemical processes.

The above discussion glosses over many details relating to photochemical processes, including the importance of a thermodynamically favorable “power stroke” in photochemical NESS, which is irrelevant in the case of chemically driven systems.^17a,52^ Indeed, we note that light-driven processes accord more closely with macroscopic expectations conjured by the word “fuel”—as well as their reliance on a power stroke, we can clearly identify steps in which energy is added and where it is stored and a photo-driven cycle forced to run backward would not emit photons. Thus, using “fuel” to describe both light- and chemical-driven systems seems problematic.

These points notwithstanding, our main intention is to highlight that, as with chemically driven systems, the key is whether the photochemical process is coupled to the process of interest or not? If it is not, then this part of the system is unaffected and “at equilibrium”.

## Conclusions

We hope that by juxtaposing the common features of catalytic systems, autonomous molecular motors, and self-assembled systems that achieve a NESS we will inspire discussion about these related exciting areas. By consistently focusing on the coupled reaction and how it interacts with the reaction network, we hope to shift the attention onto this key feature of all these systems, as well as the requirements that must be met (spontaneous process, kinetic asymmetry, reaction coupled to the process of interest) to achieve a NESS. These requirements are already well established through the work of Astumian;^13^ one of our goals is that the analysis he advocates be applied accurately in these related areas. We have also noted that unambiguously demonstrating that a system achieves a NESS is much easier in catalytic systems (simple observable) than in molecular motors (proven indirectly) and supramolecular materials (requires detailed characterization of the network).

We have also presented our concerns about some of the terminology currently used in the field. It seems that since many practitioners are inspired by biological processes, some language used in biochemistry has been adopted uncritically. An example is the focus in biology on the chemically incorrect, in our view, concept of ATP acting as a fuel, and the related and definitely incorrect concept of reactions being driven by “high-energy” bonds.^50^ This section is intentionally provocative, as we hope to encourage robust discussion. We propose the use of chemically “driven” instead of “fueled” for coupled systems that meet the requirements for a NESS, as this term does not have other connotations and associations. Similarly, we suggest that the words “fuel” and “waste” be replaced by “substrate” and “product”, respectively.

Even more importantly, “fueled” or “driven” must be reserved for systems in which the spontaneous reaction is coupled to the process of interest such that this part of the network achieves NESS; it is clearly inappropriate to use the term “fueled” self-assembly if the aggregated state is not actually coupled to the “fuel–waste” reaction. Indeed, we note that we are not alone in thinking there are currently linguistic problems; Prins proposed the terms “assembly under dissipative conditions” and “dissipative self-assembly” to differentiate materials whose assembly is influenced by a spontaneous process but not coupled to it and those in which direct coupling takes place (e.g., Figure 9c and Figure 9b, respectively), with the latter termed “driven” if kinetic asymmetry is present.^11b,12a^ However, we reiterate that, in our view, the focus on dissipation is unhelpful.

The study of molecular motors or supramolecular materials that require a NESS to achieve their function provides a way to try to understand the operation of similar systems in biology, where they are ubiquitous. Conversely, their ubiquity in biological systems is often used to justify the potential importance of synthetic non-equilibrium systems.^10−12^ It is undoubtedly true that chemically driven networks allow some behaviors, such as continuous operation of molecular motors, that are not possible by switching between equilibrium steady states. However, as these related fields mature, it is important to start to consider where they might have the greatest impact—it is not obvious that chemists will use synthetic systems for the same reasons and in the same way as they are used in biology. For example, biological molecular motors are required to operate continuously without user intervention. The same is not true of artificial systems, where energy ratchets may be simpler to apply. Put more bluntly, although both birds and rockets fly, the inspiration for the Apollo program did not come from biology—our imagination can and should supersede its example.

This is not to say that the design and operation of new systems that can achieve a NESS should be judged on the basis of their applications. In the context of molecular motors, very few working systems have been reported, and many questions remain to be answered^52^ (e.g., how to optimize systems to work against load and maximize efficiency)^62^ and challenges overcome (how to couple directional motion to the wider system to do work or achieve other objectives). Similarly, in the case of supramolecular self-assembly, we have highlighted that it remains challenging to even determine if formation of the aggregated state is actually coupled to the driving chemical reaction, let alone to articulate ahead of this what the potential benefits of achieving such behavior might be. Furthermore, although it is tempting to look to biology for the potential benefits of such systems, as we note in the context of actin, the behavior of biopolymers *in vivo* is extremely complicated. Indeed, tubulin assembly,^45^ which has some similarities to actin assembly and is also sometimes cited as inspiration for synthetic efforts,^11^ is not even as well understood as actin; the most striking features of tubulin behavior (e.g., rescue and catastrophe) are acknowledged by the biophysical community to be “complex and poorly understood”.^45c^

Thus, the final aim of this Perspective is to demystify the technical requirements for achieving non-equilibrium steady states using chemist-friendly language and notation and so to inspire chemists from across a range of areas to imagine how non-equilibrium steady states might be used in the longer term. Undoubtedly there will be exciting properties and applications, but perhaps there is a need to look beyond biology to identify them. Birds, after all, do not fly to the moon!

## Supporting Information Available

Additional discussion and derivation of equations (PDF)
