# Supplementary material for: Non-equilibrium Steady States in Catalysis, Molecular Motors, and Supramolecular Materials: Why Networks and Language Matter
Source: J Am Chem Soc. 2023 Jun 21;145(26):14169–83. doi: 10.1021/jacs.2c12665 (PMC10326876; doi:10.1021/jacs.2c12665)
Supplement: Supplementary file 1 — ja2c12665_si_001.pdf [file ja2c12665_si_001.pdf]

Non-Equilibrium Steady States in Catalysis, Molecular Motors and Supramolecular Materials: Why Networks and Language Matter

Ivan Aprahamian,*[a] Stephen M. Goldup*[b]

[a] 6128 Burke Laboratory, Department of Chemistry, Dartmouth College, Hanover, New Hampshire 03755, USA.

[b] School of Chemistry, University of Birmingham, Birmingham, B15 2TT, UK.

S. M. Goldup: s.m.goldup@bham.ac.uk
I. Aprahamian: ivan.aprahamian@dartmouth.edu

Contents

General comments 3

1. Simple ester hydrolysis networks 3

1.1a General solution for coupling A→B to X→Y using trajectory thermodynamics 3

1.1b Ester hydrolysis cycle using trajectory thermodynamics 4

1.1c Ester hydrolysis cycle using the chemical network approach 5

1.1d Flux within the simple ester hydrolysis network...6

1.1e Comparison between the ester hydrolysis network composed of elementary steps ($B_{Al}2$ mechanism) and the expanded network in which hydrolysis takes place via a tetrahedral intermediate ($B_{AC}2$ mechanism)..................6

1.2 Ester hydrolysis network in which RCO_2Me→RCO_2^- is coupled to MeI hydrolysis but R`CO_2Me is not............8

1.2a Is exchange between RCO_2Me and R`CO_2Me perturbed by the coupled reaction?.......................................8

1.2b How does the inclusion of the exchange between RCO_2Me and R`CO_2Me affect the ester hydrolysis cycle?8

1.3 Network where the hydrolysis of both ester conformers is coupled to MeI hydrolysis 8

1.3a Deriving the ratcheting constant, r_0, for the cyclic ester hydrolysis network 8

1.3b Relative concentrations of species in the cyclic ester hydrolysis network at steady state...........................10

2. Minor Enantiomer Recycling 12

2.1 How does coupling acyl cyanohydrin formation to acyl cyanide hydrolysis produce a non-equilibrium steady state? 12

2.2 What conditions must be met to observe an *ee* in using the MER strategy? 12

2.3 Selectivity in an MER system: 13

2.4 Iterative MER – reinforcement of *ee* by independent catalysts...13

3. Operation of catenane 6...15

3.1 Derivation of the ratchetting constant for catenane 6[2] 15

3.2 Relationship between the free energy change associated with mass action and the directionality of 6........16

3.3 Relationship between the directionality of 6 and the maximum work that can be performed 17

4. Free energy changes and maximum work of energy ratchets – catenane 11^+ 18

4.1 Maximum work of catenane 11 under conditions of quantitative protonation/deprotonation 18

4.2 Maximum work of catenane 11 under conditions of where protonation/deprotonation are not quantitative 19

General comments

The purpose of this document is to provide derivations of equations used and support statements made in the main text. We note that the vast majority of what follows is based on previous work from Astumian and others and this will be cited where relevant. Throughout we will use standard notation used by chemists when discussing reaction kinetics (concentrations, rate constants, equilibrium constants, activation energies etc.), which will hopefully ensure that the equations presented are accessible to the wider chemistry community.

1. Simple ester hydrolysis networks

1.1a General solution for coupling A→B to X→Y using trajectory thermodynamics[1]

At equilibrium steady state, for every molecule of **A** that is converted to **B** per unit time, one molecule of **B** is converted to **A**. We shall consider how the concentrations of **A** and **B** are affected if their exchange is "coupled" to the exchange of **X** and **Y** (**Scheme S1a**), and the concentrations of **X** and **Y** are chemostated away from their equilibrium values. To construct the required equations, we consider that all transitions can be coupled (*i.e.*, we are unbiased in deciding which steps are chemically feasible), which provides a new set of potential chemical processes that exchange **A** and **B** (**Scheme S1b**).

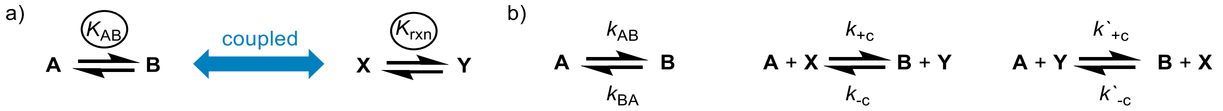

Scheme S1. a) the reactions to be coupled. b) Processes that can nominally exchange **A** and **B** if they are coupled in an unbiased manner.

At steady state, $\frac{d[\mathbf{A}]}{dt} = 0$, which allows us to write an equation involving the possible transitions in the system:

$$\frac{\mathrm{d}[A]}{\mathrm{dt}} = 0 = -k_{\mathrm{AB}}[A] - k_{+\mathrm{c}}[\mathbf{A}][\mathbf{X}] - k'_{+\mathrm{c}}[\mathbf{A}][\mathbf{Y}] + k_{\mathrm{BA}}[\mathbf{B}] + k_{-\mathrm{c}}[\mathbf{B}][\mathbf{Y}] + k'_{-\mathrm{c}}[\mathbf{B}][\mathbf{X}]$$

From here we can rearrange to yield an expression for $\left.\frac{[\mathbf{A}]}{[\mathbf{B}]}\right|_{SS}$:

$$k_{\mathrm{AB}}[\mathbf{A}] + k_{+\mathrm{c}}[\mathbf{A}][\mathbf{X}] + k'_{+\mathrm{c}}[\mathbf{A}][\mathbf{Y}] = k_{\mathrm{BA}}[\mathbf{B}] + k_{-\mathrm{c}}[\mathbf{B}][\mathbf{Y}] + k'_{-\mathrm{c}}[\mathbf{B}][\mathbf{X}]$$

$$\Longrightarrow \left.\frac{[\mathbf{A}]}{[\mathbf{B}]}\right|_{\mathrm{SS}} = \frac{\mathrm{k_{BA}} + \mathrm{k_{-c}}[\mathbf{Y}] + \mathrm{k'_{-c}}[\mathbf{X}]}{\mathrm{k_{AB}} + \mathrm{k_{+c}}[\mathbf{X}] + \mathrm{k'_{+c}}[\mathbf{Y}]}$$

To make this equation useful we rearrange to a form that compares $\left.\frac{[\mathbf{A}]}{[\mathbf{B}]}\right|_{SS}$ to the equilibrium constant K_{AB} by taking $\frac{k_{\mathrm{BA}}}{k_{\mathrm{AB}}}$ as a factor in the numerator:

$$\left.\frac{[\mathbf{A}]}{[\mathbf{B}]}\right|_{\mathrm{SS}} = \frac{1}{K_{\mathrm{AB}}}\left(\frac{k_{\mathrm{AB}} + \frac{k_{-\mathrm{c}}k_{\mathrm{AB}}}{k_{\mathrm{BA}}}[\mathbf{Y}] + \frac{k'_{-\mathrm{c}}k_{\mathrm{AB}}}{k_{\mathrm{BA}}}[\mathbf{X}]}{k_{\mathrm{AB}} + k_{+\mathrm{c}}[\mathbf{X}] + k'_{+\mathrm{c}}[\mathbf{Y}]}\right)$$

To compare the denominator and numerator in the brackets, we can take factors of $k_{+c}[\mathbf{X}]$ (second term) and $k'_{-\mathrm{c}}[\mathbf{Y}]$ (third term):

$$\left.\frac{[\mathbf{A}]}{[\mathbf{B}]}\right|_{\mathrm{SS}} = \frac{1}{K_{\mathrm{AB}}}\left(\frac{k_{\mathrm{AB}} + k_{+\mathrm{c}}[\mathbf{X}]\left\{\frac{k_{-\mathrm{c}}k_{\mathrm{AB}}[\mathbf{Y}]}{k_{+\mathrm{c}}k_{\mathrm{BA}}[\mathbf{X}]}\right\} + k'_{+\mathrm{c}}[\mathbf{Y}]\left\{\frac{k'_{-\mathrm{c}}k_{\mathrm{AB}}[\mathbf{X}]}{k'_{+\mathrm{c}}k_{\mathrm{BA}}[\mathbf{Y}]}\right\}}{\mathrm{k_{AB}} + \mathrm{k_{+c}}[\mathbf{X}] + \mathrm{k'_{+c}}[\mathbf{Y}]}\right)$$

Finally, by recognising that $\frac{k_{+c}}{k_{-c}} = K_{AB}K_{XY} = \frac{k_{AB}}{k_{BA}}K_{XY}$ and, similarly, $\frac{k'_{-c}}{k'_{+c}} = \frac{K_{XY}}{K_{AB}} = \frac{k_{BA}}{k_{AB}}K_{XY}$, we get:

$$\left.\frac{[\mathbf{A}]}{[\mathbf{B}]}\right|_{SS} = \frac{1}{K_{AB}}\left(\frac{k_{AB}+k_{+c}[\mathbf{X}]\left\{\frac{1}{K_{XY}}\frac{[\mathbf{Y}]}{[\mathbf{X}]}\right\}+k'_{+c}[\mathbf{Y}]\left\{K_{XY}\frac{[\mathbf{X}]}{[\mathbf{Y}]}\right\}}{k_{AB}+k_{+c}[\mathbf{X}]+k'_{+c}[\mathbf{Y}]}\right) \quad \text{eq. S1}$$

From the form of eq. S1, even without any chemical detail, we have demonstrated that coupling these reactions can distort [**A**] and [**B**] only if:

1) $\frac{[\mathbf{Y}]}{[\mathbf{X}]} \neq K_{XY}$ → the concentrations of **X** and **Y** must not conform to their equilibrium values; the X→Y reaction must be spontaneous in either direction.

2) $\frac{k_{+c}}{k'_{+c}} \neq K_{XY}$ → the system displays what Astumian has termed "kinetic asymmetry".[2]

1.1b Ester hydrolysis cycle using trajectory thermodynamics

Trajectory thermodynamics[1] requires that, in the first instance, we simply "couple" the MeI hydrolysis reaction to the ester hydrolysis reaction (**Scheme S2**) in an unbiased manner to generate two additional pathways for ester/carboxylate exchange:

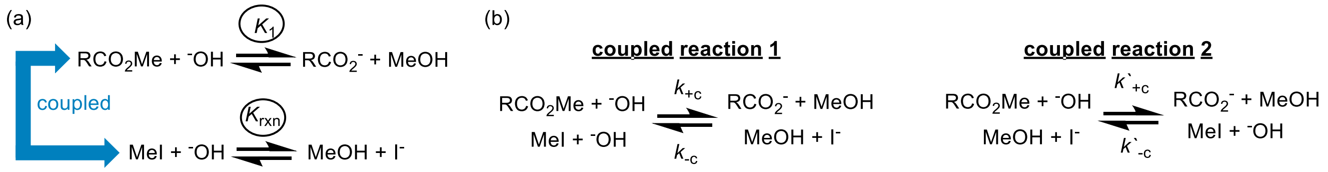

Scheme S2. a) The reactions to be coupled. b) New processes that can arise if these processes are coupled in an unbiased manner.

We note that coupled reaction 1 looks chemically unrealistic (it appears to be both reactions happening independently) and that, as drawn, coupled reaction 2 has equivalent species (MeOH and $^-$OH) on both sides of the equilibrium. However, at this stage we assume no chemical knowledge to generate suitable equations, which we can then interrogate chemically at the end. Thus, we assume reaction 1 can take place and that $^-$OH and MeOH may be catalytic in reaction 2 (taken into account by raising [$^-$OH] and [MeOH] to the power *n* in coupled reaction 2 (*n* = 0 for non-catalytic role; *n* = 1 for catalytic role)).

Given that there is no change in [RCO_2Me] at steady state:

$$\frac{d[RCO_2Me]}{dt} = 0 = -k_{+1}[RCO_2Me][OH] - k_{+c}[RCO_2Me][OH]^2[MeI] - k'_{+c}[RCO_2Me][I][OH]^n[MeOH]^n + k_{-1}[RCO_2][MeOH] + k_{-c}[RCO_2][MeOH]^2[I] + k'_{-c}[RCO_2][MeI][OH]^n[MeOH]^n$$

$$\Rightarrow [RCO_2Me](k_{+1}[OH] + k_{+c}[OH]^2[MeI] + k'_{+c}[I][OH]^n[MeOH]^n) = [RCO_2](k_{-1}[MeOH] + k_{-c}[MeOH]^2[I] + k'_{-c}[MeI][OH]^n[MeOH]^n)$$

$$\Rightarrow \left.\frac{[RCO_2Me]}{[RCO_2]}\right|_{SS} = \frac{k_{-1}[MeOH]+k_{-c}[MeOH]^2[I]+k'_{-c}[MeI][OH]^n[MeOH]^n}{k_{+1}[OH]+k_{+c}[OH]^2[MeI]+k'_{+c}[I][OH]^n[MeOH]^n}$$

To allow $\left.\frac{[RCO_2Me]}{[RCO_2]}\right|_{SS}$ to be compared to the equilibrium value, we take $\frac{[MeOH]}{[OH]}$ and $\frac{k_{-1}}{k_{+1}} = \frac{1}{K_1}$ as factors of the numerator:

$$\left.\frac{[RCO_2Me]}{[RCO_2]}\right|_{SS} = \frac{1}{K_1}\frac{[MeOH]}{[OH]}\left[\frac{k_{+1}[OH]+\frac{k_{-c}k_{+1}}{k_{-1}}[MeOH][OH][I]+\frac{k'_{-c}k_{+1}}{k_{-1}}[MeI][OH]^{n+1}[MeOH]^{n-1}}{k_{+1}[OH]+k_{+c}[OH]^2[MeI]+k'_{+c}[I][OH]^n[MeOH]^n}\right]$$

We then take $k_{+c}[\mathrm{OH}]^2[\mathrm{MeI}]$ as a factor in the 2nd term of the numerator and $k'_{+c}[\mathrm{I}][\mathrm{OH}]^n[\mathrm{MeOH}]^n$ as a factor from the 3rd term:

$$\left.\frac{[\mathrm{RCO_2Me}]}{[\mathrm{RCO_2}]}\right|_{\mathrm{SS}} = \frac{1}{K_1}\frac{[\mathrm{MeOH}]}{[\mathrm{OH}]}\left[\frac{k_{+1}[\mathrm{OH}]+k_{+c}[\mathrm{OH}]^2[\mathrm{MeI}]\left\{\frac{k_{-c}k_{+1}[\mathrm{MeOH}][\mathrm{I}]}{k_{+c}k_{-1}[\mathrm{OH}][\mathrm{MeI}]}\right\}+k'_{+c}[\mathrm{I}][\mathrm{OH}]^{\mathrm{n}}[\mathrm{MeOH}]^{\mathrm{n}}\left\{\frac{k'_{-c}k_{+1}[\mathrm{MeI}][\mathrm{OH}]}{k'_{+c}k_{-1}[\mathrm{MeOH}][\mathrm{I}]}\right\}}{k_{+1}[\mathrm{OH}]+k_{+c}[\mathrm{OH}]^2[\mathrm{MeI}]+k'_{+c}[\mathrm{I}][\mathrm{OH}]^{\mathrm{n}}[\mathrm{MeOH}]^{\mathrm{n}}}\right]$$

Finally, we recognise that $\frac{k_{-c}k_{+1}}{k_{+c}k_{-1}} = \frac{1}{K_{\mathrm{rxn}}}$ and $\frac{k'_{-c}k_{+1}}{k'_{+c}k_{-1}} = K_{\mathrm{rxn}}$ to generate eq. S3:

$$\left.\frac{[\mathrm{RCO_2Me}]}{[\mathrm{RCO_2}]}\right|_{\mathrm{SS}} = \frac{1}{K_1}\frac{[\mathrm{MeOH}]}{[\mathrm{OH}]}\left[\frac{k_{+1}[\mathrm{OH}]+k_{+c}[\mathrm{OH}]^2[\mathrm{MeI}]\left\{\frac{1}{K_{\mathrm{rxn}}}\frac{[\mathrm{MeOH}][\mathrm{I}]}{[\mathrm{OH}][\mathrm{MeI}]}\right\}+k'_{+c}[\mathrm{I}][\mathrm{OH}]^{\mathrm{n}}[\mathrm{MeOH}]^{\mathrm{n}}\left\{K_{\mathrm{rxn}}\frac{[\mathrm{MeI}][\mathrm{OH}]}{[\mathrm{MeOH}][\mathrm{I}]}\right\}}{k_{+1}[\mathrm{OH}]+k_{+c}[\mathrm{OH}]^2[\mathrm{MeI}]+k'_{+c}[\mathrm{I}][\mathrm{OH}]^{\mathrm{n}}[\mathrm{MeOH}]^{\mathrm{n}}}\right] \qquad \text{eq. S2}$$

Eq. S2 has exactly the same form as eq. S1, and so equivalent requirements must be met for achieving a non-equilibrium steady state:

1) $\frac{[\mathrm{MeOH}][\mathrm{I}]}{[\mathrm{OH}][\mathrm{MeI}]} \neq K_{\mathrm{rxn}}$ → the concentrations of the species involved in the coupled reaction must not conform to their equilibrium values; the coupled reaction must be spontaneous in either direction.

2) $\frac{k_{+c}}{k'_{+c}} \neq K_{\mathrm{rxn}}$ → the system displays kinetic asymmetry.

Of course, because this is a real system, we can recognise that coupled reaction 1 is not chemically realistic (it represents both reactions happening independently) and so k_{+c} (and k_{-c}) can be set to 0. Thus, kinetic asymmetry is automatically a feature of this network once the chemical detail is included. We can also recognise the $^{-}$OH and MeOH are not catalytic in coupled reaction 2 (which represents the reaction of $\mathrm{I^-}$ with the ester) and so n = 0, yielding eq. S3:

$$\left.\frac{[\mathrm{RCO_2Me}]}{[\mathrm{RCO_2}]}\right|_{\mathrm{SS}} = \frac{1}{K_1}\frac{[\mathrm{MeOH}]}{[\mathrm{OH}]}\left[\frac{k_{+1}[\mathrm{OH}]+k'_{+c}[\mathrm{I}]\left\{K_{\mathrm{rxn}}\frac{[\mathrm{MeI}][\mathrm{OH}]}{[\mathrm{MeOH}][\mathrm{I}]}\right\}}{k_{+1}[\mathrm{OH}]+k'_{+c}[\mathrm{I}]}\right] \qquad \text{eq. S3}$$

1.1c Ester hydrolysis cycle using the chemical network approach

We can follow the same process starting from the chemical network involving ester hydrolysis and ester formation by reaction with MeI (**Scheme S3**):

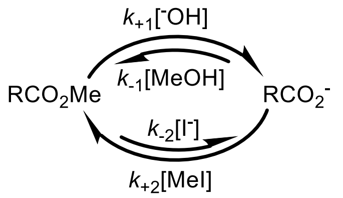

Scheme S3. Reaction network established when ester hydrolysis takes place in the presence of MeI.

Using the fact that there is no change in [RCO_2Me] at steady state we can generate an expression for $\left.\frac{[\mathrm{RCO_2Me}]}{[\mathrm{RCO_2}]}\right|_{SS}$:

$$\frac{\mathrm{d}[\mathrm{RCO_2Me}]}{\mathrm{dt}} = 0 = -k_{+1}[\mathrm{RCO_2Me}][\mathrm{OH}] - k_{-2}[\mathrm{RCO_2Me}][\mathrm{I}] + k_{-1}[\mathrm{RCO_2}][\mathrm{MeOH}] + k_{+2}[\mathrm{RCO_2}][\mathrm{MeI}]$$

$$\Longrightarrow k_{+1}[\mathrm{RCO_2Me}][\mathrm{OH}] + k_{-2}[\mathrm{RCO_2Me}][\mathrm{I}] = k_{-1}[\mathrm{RCO_2}][\mathrm{MeOH}] + k_{+2}[\mathrm{RCO_2}][\mathrm{MeI}]$$

$$\Longrightarrow \left.\frac{[\mathrm{RCO_2Me}]}{[\mathrm{RCO_2}]}\right|_{\mathrm{SS}} = \frac{k_{-1}[\mathrm{MeOH}]+k_{+2}[\mathrm{MeI}]}{k_{+1}[\mathrm{OH}]+k_{-2}[\mathrm{I}]}$$

As before, we can then rearrange to compare $\left.\frac{[\mathrm{RCO_2Me}]}{[\mathrm{RCO_2}]}\right|_{SS}$ with the corresponding equilibrium steady state:

$$\left.\frac{[\mathrm{RCO_2Me}]}{[\mathrm{RCO_2}]}\right|_{\mathrm{SS}} = \frac{1}{K_1}\frac{[\mathrm{MeOH}]}{[\mathrm{OH}]}\left[\frac{k_{+1}[\mathrm{OH}]+\frac{k_{+2}k_{+1}}{k_{-1}}\frac{[\mathrm{MeI}][\mathrm{OH}]}{[\mathrm{MeOH}]}}{k_{+1}[\mathrm{OH}]+k_{-2}[\mathrm{I}]}\right]$$

Finally, recognising that $\frac{k_{+2}k_{+1}}{k_{-2}k_{-1}} = K_{rxn}$ allows us to generate eq. S4:

$$\left.\frac{[\mathrm{RCO_2Me}]}{[\mathrm{RCO_2}]}\right|_{\mathrm{SS}} = \frac{1}{K_1}\frac{[\mathrm{MeOH}]}{[\mathrm{OH}]}\left[\frac{k_{+1}[\mathrm{OH}]+k_{-2}[\mathrm{I}]\left\{K_{\mathrm{rxn}}\frac{[\mathrm{MeI}][\mathrm{OH}]}{[\mathrm{MeOH}][\mathrm{I}]}\right\}}{k_{+1}[\mathrm{OH}]+k_{-2}[\mathrm{I}]}\right] \qquad \text{eq. S4}$$

Eq. S4 has exactly the same form as eq. S3 where $k'_{+\mathrm{c}} = k_{-2}$. Thus, the chemical network approach is equivalent to the trajectory thermodynamics approach. The latter has the advantage of avoiding introducing bias early in the construction of the mathematical model, but the former is more intuitive for a chemist.

1.1d Flux within the simple ester hydrolysis network

The rate of flux in a chemical cycle can be quantified by the ratcheting constant, r_0 (eq. S5):[2]

$$r_0 = \frac{\text{rate of forward step 1}\times\text{rate of forward step 2}\times\ldots}{\text{rate of reverse step 1}\times\text{rate of reverse step 2}\times\ldots} \qquad \text{eq. S5}$$

Applying this equation in the ester hydrolysis network yields:

$$r_0 = \frac{k_{+2}[\mathrm{MeI}][\mathrm{RCO_2}]\times k_{+1}[\mathrm{OH}][\mathrm{RCO_2Me}]}{k_{-2}[\mathrm{I}][\mathrm{RCO_2Me}]\times k_{-1}[\mathrm{MeOH}][\mathrm{RCO_2}]}$$

$$\Rightarrow r_0 = \left\{\frac{k_{+1}k_{+2}}{k_{-1}k_{-2}}\right\}\left\{\frac{[\mathrm{MeI}][\mathrm{OH}]}{[\mathrm{I}][\mathrm{MeOH}]}\right\}$$

$$\Rightarrow r_0 = K_{\mathrm{rxn}}\left\{\frac{[\mathrm{MeI}][\mathrm{OH}]}{[\mathrm{I}][\mathrm{MeOH}]}\right\} \qquad \text{eq. S6}$$

From the form of eq. S6, we can see that there will be net flux over the two different transition states that connect RCO_2^- and RCO_2Me if the coupled reaction is spontaneous.

1.1e Comparison between the ester hydrolysis network composed of elementary steps ($B_{Al}2$ mechanism) and the expanded network in which hydrolysis takes place via a tetrahedral intermediate ($B_{Ac}2$ mechanism)

To confirm that including a two-step hydrolysis (**Scheme S4b**) does not alter the conclusions drawn using the simple one-step pathway (**Scheme S4a**) we can compare the forms of the ratcheting constant and $\left.\frac{[\mathrm{RCO_2Me}]}{[\mathrm{RCO_2}]}\right|_{SS}$ in each case.

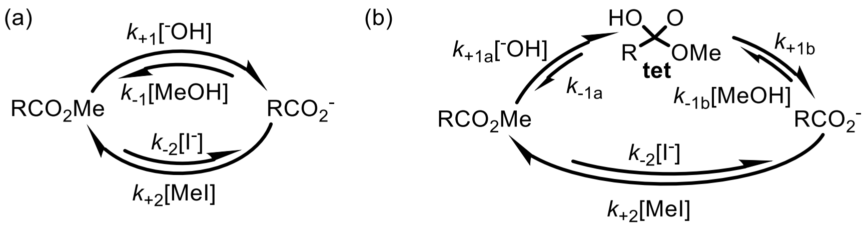

Scheme S4. Comparison between the networks established when the hydrolysis reaction is (a) single step ($B_{Al}2$) and (b) two step ($B_{Ac}2$).

It is straightforward to confirm that the form of r_0 is identical to that obtained in the simple network (eq. S6):

$$r_0 = \frac{(k_{+2}[\mathrm{RCO_2}][\mathrm{MeI}])\times(k_{+1a}[\mathrm{RCO_2Me}][\mathrm{OH}])\times(k_{+1b}[\mathrm{tet}])}{(k_{-1b}[\mathrm{RCO_2}][\mathrm{MeOH}])\times(k_{-1a}[\mathrm{tet}])\times(k_{-2}[\mathrm{RCO_2Me}][\mathrm{I}])} = \frac{k_{+2}k_{+1a}k_{+1b}}{k_{-1b}k_{-1a}k_{-2}}\frac{[\mathrm{MeI}][\mathrm{OH}]}{[\mathrm{MeOH}][\mathrm{I}]} = K_{\mathrm{rxn}}\left\{\frac{[\mathrm{MeI}][\mathrm{OH}]}{[\mathrm{I}][\mathrm{MeOH}]}\right\}$$

To evaluate $\left.\frac{[\mathrm{RCO_2Me}]}{[\mathrm{RCO_2}]}\right|_{SS}$, we first generate simple expressions for $\frac{d[\mathrm{RCO_2Me}]}{dt}$ and $\frac{d[\mathrm{RCO_2}]}{dt}$ at steady state:

$$\frac{d[\mathrm{RCO_2Me}]}{dt} = 0 = -k_{+1\mathrm{a}}[\mathrm{RCO_2Me}][\mathrm{OH}] - k_{-2}[\mathrm{RCO_2Me}][\mathrm{I}] + k_{-1\mathrm{a}}[\mathrm{tet}] + \mathrm{k}_{+2}[\mathrm{RCO_2}][\mathrm{MeI}]$$

$$\Longrightarrow k_{-1\mathrm{a}}[\mathrm{tet}] = k_{+1\mathrm{a}}[\mathrm{RCO_2Me}][\mathrm{OH}] + k_{-2}[\mathrm{RCO_2Me}][\mathrm{I}] - k_{+2}[\mathrm{RCO_2}][\mathrm{MeI}]$$

$$\frac{d[\mathrm{RCO_2}]}{dt} = 0 = -k_{+2}[\mathrm{RCO_2}][\mathrm{MeI}] + k_{-2}[\mathrm{RCO_2Me}][\mathrm{I}] + k_{+1\mathrm{b}}[\mathrm{tet}] - k_{-1\mathrm{b}}[\mathrm{RCO_2}][\mathrm{MeOH}]$$

$$\Longrightarrow k_{+1\mathrm{b}}[\mathrm{tet}] = k_{+2}[\mathrm{RCO_2}][\mathrm{MeI}] + k_{-1\mathrm{b}}[\mathrm{RCO_2}][\mathrm{MeOH}] - k_{-2}[\mathrm{RCO_2Me}][\mathrm{I}]$$

Dividing these expressions by one another to eliminate [tet] yields eq. S7:

$$\frac{k_{-1\mathrm{a}}}{k_{+1\mathrm{b}}} = \frac{k_{+1\mathrm{a}}[\mathrm{RCO_2Me}][\mathrm{OH}]+k_{-2}[\mathrm{RCO_2Me}][\mathrm{I}]-k_{+2}[\mathrm{RCO_2}][\mathrm{MeI}]}{k_{+2}[\mathrm{RCO_2}][\mathrm{MeI}]+k_{-1\mathrm{b}}[\mathrm{RCO_2}][\mathrm{MeOH}]-k_{-2}[\mathrm{RCO_2Me}][\mathrm{I}]} \qquad \text{eq. S7}$$

Eq. S7 can be rearranged to gather terms in $[\mathrm{RCO_2Me}]$ and $[\mathrm{RCO_2}]$ leading to an expression for $\left.\frac{[\mathrm{RCO_2Me}]}{[\mathrm{RCO_2}]}\right|_{SS}$:

$$\text{eq. 7} \Longrightarrow k_{-1\mathrm{a}}[\mathrm{RCO_2}](\mathrm{k}_{+2}[\mathrm{MeI}] + k_{-1\mathrm{b}}[\mathrm{MeOH}]) - k_{-1\mathrm{a}}k_{-2}[\mathrm{RCO_2Me}][\mathrm{I}] = k_{+1\mathrm{b}}[\mathrm{RCO_2Me}](\mathrm{k}_{+1\mathrm{a}}[\mathrm{OH}] + k_{-2}[\mathrm{I}]) - k_{+1\mathrm{b}}k_{+2}[\mathrm{RCO_2}][\mathrm{MeI}]$$

$$\Longrightarrow k_{-1\mathrm{a}}[\mathrm{RCO_2}]\left(k_{+2}[\mathrm{MeI}] + k_{-1\mathrm{b}}[\mathrm{MeOH}] + \frac{k_{+1\mathrm{b}}k_{+2}}{k_{-1\mathrm{a}}}[\mathrm{MeI}]\right) = k_{+1\mathrm{b}}[\mathrm{RCO_2Me}]\left(k_{+1\mathrm{a}}[\mathrm{OH}] + k_{-2}[\mathrm{I}] + \frac{k_{-1\mathrm{a}}k_{-2}}{k_{+1\mathrm{b}}}[\mathrm{I}]\right)$$

$$\Longrightarrow \left.\frac{[\mathrm{RCO_2Me}]}{[\mathrm{RCO_2}]}\right|_{SS} = \frac{k_{-1\mathrm{a}}}{k_{+1\mathrm{b}}}\left\{\frac{k_{+2}[\mathrm{MeI}]+k_{-1\mathrm{b}}[\mathrm{MeOH}]+\frac{k_{+1\mathrm{b}}k_{+2}}{k_{-1\mathrm{a}}}[\mathrm{MeI}]}{k_{+1\mathrm{a}}[\mathrm{OH}]+k_{-2}[\mathrm{I}]+\frac{k_{-1\mathrm{a}}k_{-2}}{k_{+1\mathrm{b}}}[\mathrm{I}]}\right\} = \frac{k_{-1\mathrm{a}}}{k_{+1\mathrm{b}}}\left\{\frac{k_{-1\mathrm{b}}[\mathrm{MeOH}]+k_{+2}[\mathrm{MeI}]\left(1+\frac{k_{+1\mathrm{b}}}{k_{-1\mathrm{a}}}\right)}{k_{+1\mathrm{a}}[\mathrm{OH}]+k_{-2}[\mathrm{I}]\left(1+\frac{k_{-1\mathrm{a}}}{k_{+1\mathrm{b}}}\right)}\right\}$$

Taking $\frac{k_{-1\mathrm{b}}}{k_{+1\mathrm{a}}}\frac{[\mathrm{MeOH}]}{[\mathrm{OH}]}$ as a factor from the numerator yields:

$$\left.\frac{[\mathrm{RCO_2Me}]}{[\mathrm{RCO_2}]}\right|_{SS} = \frac{k_{-1\mathrm{a}}k_{-1\mathrm{b}}}{k_{+1\mathrm{b}}k_{+1\mathrm{a}}}\frac{[\mathrm{MeOH}]}{[\mathrm{OH}]}\left\{\frac{k_{+1\mathrm{a}}[\mathrm{OH}]+\frac{k_{+1\mathrm{a}}k_{+2}}{k_{-1\mathrm{b}}}\frac{[\mathrm{MeI}][\mathrm{OH}]}{[\mathrm{MeOH}]}\left(1+\frac{k_{+1\mathrm{b}}}{k_{-1\mathrm{a}}}\right)}{k_{+1\mathrm{a}}[\mathrm{OH}]+k_{-2}[\mathrm{I}]\left(1+\frac{k_{-1\mathrm{a}}}{k_{+1\mathrm{b}}}\right)}\right\}$$

Taking $k_{-2}[\mathrm{I}]\frac{k_{+1\mathrm{b}}}{k_{-1\mathrm{a}}}$ as a factor in the 2nd term of the numerator yields:

$$\left.\frac{[\mathrm{RCO_2Me}]}{[\mathrm{RCO_2}]}\right|_{SS} = \frac{k_{-1\mathrm{a}}k_{-1\mathrm{b}}}{k_{+1\mathrm{b}}k_{+1\mathrm{a}}}\frac{[\mathrm{MeOH}]}{[\mathrm{OH}]}\left\{\frac{k_{+1\mathrm{a}}[\mathrm{OH}]+k_{-2}[\mathrm{I}]\left(1+\frac{k_{-1\mathrm{a}}}{k_{+1\mathrm{b}}}\right)\frac{k_{+1\mathrm{a}}k_{+1\mathrm{b}}k_{+2}[\mathrm{MeI}][\mathrm{OH}]}{k_{-1\mathrm{b}}k_{-1\mathrm{a}}k_{-2}[\mathrm{MeOH}][\mathrm{I}]}}{k_{+1\mathrm{a}}[\mathrm{OH}]+k_{-2}[\mathrm{I}]\left(1+\frac{k_{-1\mathrm{a}}}{k_{+1\mathrm{b}}}\right)}\right\}$$

Substituting $\frac{k_{-1\mathrm{a}}k_{-1\mathrm{b}}}{k_{+1\mathrm{b}}k_{+1\mathrm{a}}} = \frac{1}{K_1}$ where K_1 is the equilibrium constant for the overall ester/carboxylate hydrolysis equilibrium, and $\frac{k_{+1\mathrm{a}}k_{+1\mathrm{b}}k_{+2}}{k_{-1\mathrm{b}}k_{-1\mathrm{a}}k_{-2}} = K_{\mathrm{rxn}}$ yields eq. S8:

$$\left.\frac{[\mathrm{RCO_2Me}]}{[\mathrm{RCO_2}]}\right|_{SS} = \frac{1}{K_1}\frac{[\mathrm{MeOH}]}{[\mathrm{OH}]}\left\{\frac{k_{+1\mathrm{a}}[\mathrm{OH}]+k_{-2}[\mathrm{I}]\left(1+\frac{k_{-1\mathrm{a}}}{k_{+1\mathrm{b}}}\right)\left\{K_{\mathrm{rxn}}\frac{[\mathrm{MeI}][\mathrm{OH}]}{[\mathrm{MeOH}][\mathrm{I}]}\right\}}{k_{+1\mathrm{a}}[\mathrm{OH}]+k_{-2}[\mathrm{I}]\left(1+\frac{k_{-1\mathrm{a}}}{k_{+1\mathrm{b}}}\right)}\right\} \qquad \text{eq. S8}$$

Comparing eq. S8 with eq. S4, reveals they have the same form. Thus, the general conclusion that $\left.\frac{[\mathrm{RCO_2Me}]}{[\mathrm{RCO_2}]}\right|_{SS} = \frac{1}{K_1}\frac{[\mathrm{MeOH}]}{[\mathrm{OH}]}$ if $K_{\mathrm{rxn}}\frac{[\mathrm{MeI}][\mathrm{OH}]}{[\mathrm{MeOH}][\mathrm{I}]} = 1$ (i.e., the coupled reaction is at equilibrium) is identical.

1.2 Ester hydrolysis network in which $RCO_2Me \rightarrow RCO_2^-$ is coupled to MeI hydrolysis but R\`CO_2Me is not.

To examine the behaviour of the network in which only one of the ester conformers is in exchange with RCO_2^- (**Scheme S5**) we can evaluate the exchange of RCO_2Me and R\`CO_2Me, and RCO_2Me with RCO_2^-.

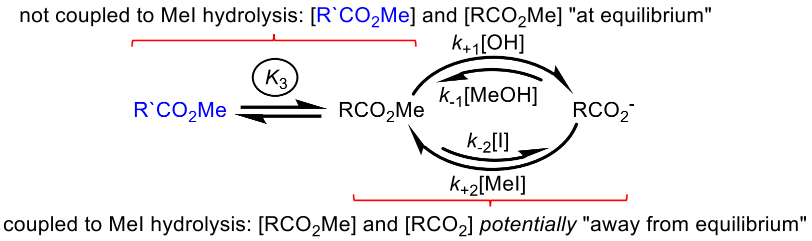

Scheme S5. Reaction network in which only some processes are coupled to the hydrolysis of MeI.

1.2a Is exchange between RCO_2Me and R\`CO_2Me perturbed by the coupled reaction?

If we focus on the exchange of RCO_2Me and R\`CO_2Me, at steady state:

$$\frac{d[\mathrm{R`CO_2Me}]}{dt} = 0 = -k_{-3}[\mathrm{R`CO_2Me}] + k_{+3}[\mathrm{RCO_2Me}]$$

Rearranging, we recover the standard expression (eq. S7) for the relative concentrations of the two conformations at equilibrium. Thus, although the overall system can achieve a non-equilibrium steady state (see below), the conformational exchange equilibrium is not affected by the coupled reaction:

$$\left.\frac{[\mathrm{R`CO_2Me}]}{[\mathrm{RCO_2Me}]}\right|_{SS} = \frac{k_{+3}}{k_{-3}} = K_3 \qquad \text{eq. S9}$$

1.2b How does the inclusion of the exchange between RCO_2Me and R\`CO_2Me affect the ester hydrolysis cycle?

Using the fact that there is no change in [RCO_2Me] at steady state:

$$\frac{d[\mathrm{RCO_2Me}]}{dt} = 0 = -k_{+1}[\mathrm{RCO_2Me}][\mathrm{OH}] - k_{-2}[\mathrm{RCO_2Me}][\mathrm{I}] - k_{+3}[\mathrm{RCO_2Me}] + k_{-1}[\mathrm{RCO_2}][\mathrm{MeOH}] + k_{+2}[\mathrm{RCO_2}][\mathrm{MeI}] + k_{-3}[\mathrm{R`CO_2Me}]$$

Using eq. S9, we can substitute $[\mathrm{R`CO_2Me}] = \frac{k_{+3}}{k_{-3}}[\mathrm{RCO_2Me}]$, which yields:

$$0 = -k_{+1}[\mathrm{RCO_2Me}][\mathrm{OH}] - k_{-2}[\mathrm{RCO_2Me}][\mathrm{I}] + k_{-1}[\mathrm{RCO_2}][\mathrm{MeOH}] + k_{+2}[\mathrm{RCO_2}][\mathrm{MeI}]$$

Since this equation is identical to the expression obtained without the additional conformational exchange (Section S1.1c), it is clear that the ester hydrolysis cycle is unaffected by the conformational exchange.

1.3 Network where the hydrolysis of both ester conformers is coupled to MeI hydrolysis

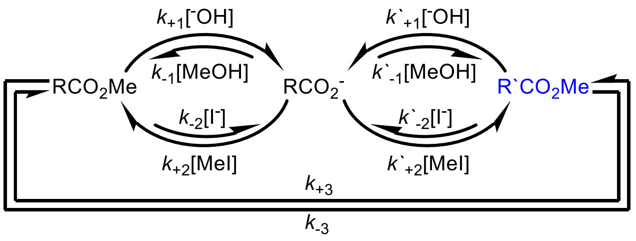

Scheme S6. Full network in which both ester conformers undergo hydrolysis/formation

1.3a Deriving the ratcheting constant, r_0, for the cyclic ester hydrolysis network

Using eq. S5 and starting from RCO_2^-, moving clockwise around the network in the numerator (**Scheme S6**), we get:

$$r_0 = \frac{(k`_{+2}[\mathrm{RCO_2}][\mathrm{MeI}]+k`_{-1}[\mathrm{RCO_2}][\mathrm{MeOH}])\times(k_{+3}[\mathrm{R`CO_2Me}])\times(k_{+1}[\mathrm{RCO_2Me}][\mathrm{OH}]+k_{-2}[\mathrm{RCO_2Me}][\mathrm{I}])}{(k_{-1}[\mathrm{RCO_2}][\mathrm{MeOH}]+k_{+2}[\mathrm{RCO_2}][\mathrm{MeI}])\times(k_{-3}[\mathrm{RCO_2Me}])\times(k`_{+1}[\mathrm{R`CO_2Me}][\mathrm{OH}]+k`_{-2}[\mathrm{R`CO_2Me}][\mathrm{I}])}$$

The concentrations of RCO_2^-, RCO_2Me and $R`CO_2Me$ cancel to give:

$$r_0 = \frac{(k`_{-1}[\mathrm{MeOH}]+k`_{+2}[\mathrm{MeI}])\times(k_{+3})\times(k_{+1}[\mathrm{OH}]+k_{-2}[\mathrm{I}])}{(k_{-1}[\mathrm{MeOH}]+k_{+2}[\mathrm{MeI}])\times(k_{-3})\times(k`_{+1}[\mathrm{OH}]+k`_{-2}[\mathrm{I}])}$$

Gathering the $k`$ and k terms yields:

$$r_0 = \frac{k_{+3}}{k_{-3}}\left[\frac{k_{+1}[\mathrm{OH}]+k_{-2}[\mathrm{I}]}{k_{-1}[\mathrm{MeOH}]+k_{+2}[\mathrm{MeI}]}\right]\left[\frac{k`_{-1}[\mathrm{MeOH}]+k`_{+2}[\mathrm{MeI}]}{k`_{+1}[\mathrm{OH}]+k`_{-2}[\mathrm{I}]}\right]$$

Taking $\frac{k_{-2}}{k_{+2}}$ as a factor of first bracket numerator and $\frac{k`_{-2}}{k`_{+2}}$ from the denominator of the second bracket yields:

$$r_0 = \frac{k_{+3}}{k_{-3}}\frac{k_{-2}}{k_{+2}}\frac{k'_{+2}}{k'_{-2}}\left[\frac{\frac{k_{+1}k_{+2}}{k_{-2}}[\mathrm{OH}]+k_{+2}[\mathrm{I}]}{k_{-1}[\mathrm{MeOH}]+k_{+2}[\mathrm{MeI}]}\right]\left[\frac{k`_{-1}[\mathrm{MeOH}]+k`_{+2}[\mathrm{MeI}]}{\frac{k`_{+1}k`_{+2}}{k`_{-2}}[\mathrm{OH}]+k`_{+2}[\mathrm{I}]}\right]$$

Recognising that $\frac{k_{+3}}{k_{-3}}\frac{k_{-2}}{k_{+2}}\frac{k`_{+2}}{k`_{-2}} = K_3K`_2K_2^{-1} \equiv 1$ (cyclic equilibria) yields eq. S10:

$$r_0 = \left[\frac{\frac{k_{+1}k_{+2}}{k_{-2}}[\mathrm{OH}]+k_{+2}[\mathrm{I}]}{k_{-1}[\mathrm{MeOH}]+k_{+2}[\mathrm{MeI}]}\right]\left[\frac{k`_{-1}[\mathrm{MeOH}]+k`_{+2}[\mathrm{MeI}]}{\frac{k`_{+1}k`_{+2}}{k`_{-2}}[\mathrm{OH}]+k`_{+2}[\mathrm{I}]}\right] = \mathrm{AB}^{-1} \qquad \text{eq. S10}$$

Labelling the first bracket from eq. S10 as "*A*", we can take $\frac{[\mathrm{I}]}{[\mathrm{MeI}]}$ as a factor from the numerator and then divide top and bottom by k_{-1} to get:

$$\mathrm{A} = \frac{[\mathrm{I}]}{[\mathrm{MeI}]}\left[\frac{\frac{k_{+1}k_{+2}}{k_{-2}k_{-1}}\frac{[\mathrm{MeI}][\mathrm{OH}]}{[\mathrm{I}]}+\frac{k_{+2}}{k_{-1}}[\mathrm{MeI}]}{[\mathrm{MeOH}]+\frac{k_{+2}}{k_{-1}}[\mathrm{MeI}]}\right]$$

Recognising that $\frac{k_{+1}k_{+2}}{k_{-2}k_{-1}} = K_{rxn}$ and taking [MeOH] as a factor in the first term yields:

$$\mathrm{A} = \frac{[\mathrm{I}]}{[\mathrm{MeI}]}\left[\frac{[\mathrm{MeOH}]\left\{K_{\mathrm{rxn}}\frac{[\mathrm{MeI}][\mathrm{OH}]}{[\mathrm{MeOH}][\mathrm{I}]}\right\}+\frac{k_{+2}}{k_{-1}}[\mathrm{MeI}]}{[\mathrm{MeOH}]+\frac{k_{+2}}{k_{-1}}[\mathrm{MeI}]}\right]$$

Following the same process with the second bracket (*B*) yields:

$$\mathrm{B} = \frac{[\mathrm{I}]}{[\mathrm{MeI}]}\left[\frac{[\mathrm{MeOH}]\left\{K_{\mathrm{rxn}}\frac{[\mathrm{MeI}][\mathrm{OH}]}{[\mathrm{MeOH}][\mathrm{I}]}\right\}+\frac{k`_{+2}}{k`_{-1}}[\mathrm{MeI}]}{[\mathrm{MeOH}]+\frac{k`_{+2}}{k`_{-1}}[\mathrm{MeI}]}\right]$$

Substituting these expressions for A and B into eq. S10 yields eq. S11:

$$r_0 = \mathrm{AB}^{-1} = \left[\frac{[\mathrm{MeOH}]\left\{K_{\mathrm{rxn}}\frac{[\mathrm{MeI}][\mathrm{OH}]}{[\mathrm{MeOH}][\mathrm{I}]}\right\}+\frac{k_{+2}}{k_{-1}}[\mathrm{MeI}]}{[\mathrm{MeOH}]+\frac{k_{+2}}{\mathrm{k}_{-1}}[\mathrm{MeI}]}\right]\left[\frac{[\mathrm{MeOH}]+\frac{k`_{+2}}{k`_{-1}}[\mathrm{MeI}]}{[\mathrm{MeOH}]\left\{K_{\mathrm{rxn}}\frac{[\mathrm{MeI}][\mathrm{OH}]}{[\mathrm{MeOH}][\mathrm{I}]}\right\}+\frac{k`_{+2}}{k`_{-1}}[\mathrm{MeI}]}\right] \qquad \text{eq. S11}$$

Based on eq. S11 we can see that $r_0 \neq 1$ (*i.e.*, there is net flux around the cycle) if the following conditions are met:

1) $K_{rxn}\frac{[\mathrm{MeI}][\mathrm{OH}]}{[\mathrm{MeOH}][\mathrm{I}]} \neq 1$ (*i.e.*, the coupled reaction is spontaneous) **AND**;

2) $\frac{k_{+2}}{k_{-1}} \neq \frac{k`_{+2}}{k`_{-1}}$ (kinetic asymmetry is present).

1.3b Relative concentrations of species in the cyclic ester hydrolysis network at steady state

To derive an expression for $\left.\frac{[\mathrm{R`CO_2Me}]}{[\mathrm{RCO_2Me}]}\right|_{SS}$ it is convenient to re-express the network (**Scheme S7a**) to group the different pathways that link $RCO_2^-$ and $RCO_2Me$ in two new kinetic constants, $\Phi$ and $\Psi$, with equivalent constants $\Phi`$ and $\Psi`$ linking $RCO_2^-$ and $R`CO_2Me$ (**Scheme S7b**). We note that the graphical form of this network, with an apparent single kinetic coefficient for each step could be misleading and re-emphasise that we are treating all reactions as reversible; grouping the terms in this way simplifies the algebra to come but does not change the form of the network in any way. Also note the different arrows used in the two representations; equilibrium arrows indicate that the forward and back transmission probabilities (rate constants) are bound by microscopic reversibility whereas the simple arrows in (b) indicate that Φ, Ψ are not.

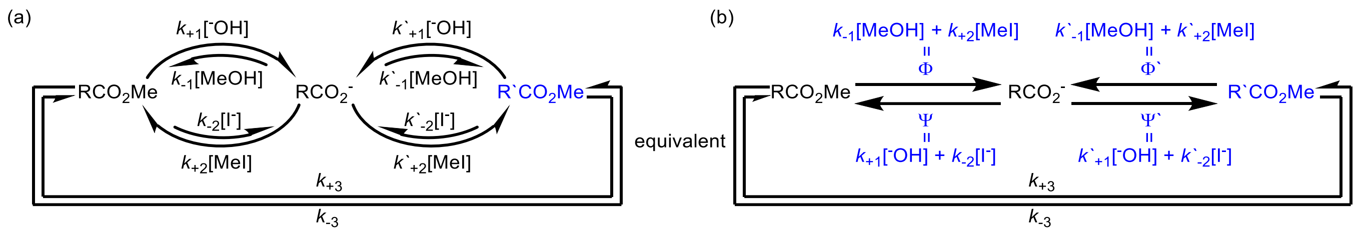

Scheme S7. (a) Full network in which both ester conformers undergo hydrolysis/formation. (b) The same network re-expressed in terms of the new kinetic constants (at fixed values of [MeOH], [MeI], [OH] and [I]) Φ, Ψ, $\Phi`$ and $\Psi`$.

Using this notation, we can re-write r_0 for this network as (eq. S12) as:

$$r_0 = \frac{(k`_{-1}[\mathrm{MeOH}]+k`_{+2}[\mathrm{MeI}])\times(k_{+3})\times(k_{+1}[\mathrm{OH}]+k_{-2}[\mathrm{I}])}{(k_{-1}[\mathrm{MeOH}]+k_{+2}[\mathrm{MeI}])\times(k_{-3})\times(k`_{+1}[\mathrm{OH}]+k`_{-2}[\mathrm{I}])} = \frac{\Psi`\mathrm{k}_{+3}\Phi}{\Psi\mathrm{k}_{-3}\Phi`} \qquad \text{eq. S12}$$

We can also use this notation to generate simple expressions for $\frac{d[\mathrm{RCO2Me}]}{dt}$ and $\frac{d[\mathrm{R`CO2Me}]}{dt}$ at steady state:

$$\frac{d[\mathrm{RCO_2Me}]}{dt} = 0 = \Psi[\mathrm{RCO_2}] - \Phi[\mathrm{RCO_2Me}] + \mathrm{k}_{+3}[\mathrm{R`CO_2Me}] - \mathrm{k}_{-3}[\mathrm{RCO_2Me}]$$

$$\frac{\mathrm{d}[\mathrm{R`CO_2Me}]}{\mathrm{dt}} = 0 = \Psi`[\mathrm{RCO_2}] - \Phi`[\mathrm{R`CO_2Me}] - k_{+3}[\mathrm{R`CO_2Me}] + k_{-3}[\mathrm{RCO_2Me}]$$

Rearranging these equations to gather terms in $[RCO_2]$ on the LHS and dividing $\Psi[\mathrm{RCO_2}]$ by $\Psi`[\mathrm{RCO_2}]$ yields:

$$\Psi[\mathrm{RCO_2}] = \Phi[\mathrm{RCO_2Me}] - k_{+3}[\mathrm{R`CO_2Me}] + k_{-3}[\mathrm{RCO_2Me}]$$

$$\Psi`[\mathrm{RCO_2}] = \Phi`[\mathrm{R`CO_2Me}] + k_{+3}[\mathrm{R`CO_2Me}] - k_{-3}[\mathrm{RCO_2Me}]$$

$$\Longrightarrow \frac{\Psi}{\Psi`} = \frac{\Phi[\mathrm{RCO_2Me}]-k_{+3}[\mathrm{R`CO_2Me}]+k_{-3}[\mathrm{RCO_2Me}]}{\Phi`[\mathrm{R`CO_2Me}]+k_{+3}[\mathrm{R`CO_2Me}]-k_{-3}[\mathrm{RCO_2Me}]}$$

Which can be rearranged as shown to yield an expression for $\left.\frac{[\mathrm{R`CO_2Me}]}{[\mathrm{RCO_2Me}]}\right|_{SS}$:

$$\Psi\Phi`[\mathrm{R`CO_2Me}] + \Psi k_{+3}[\mathrm{R`CO_2Me}] - \Psi k_{-3}[\mathrm{RCO_2Me}] = \Psi`\Phi[\mathrm{RCO_2Me}] - \Psi`k_{+3}[\mathrm{R`CO_2Me}] + \Psi`k_{-3}[\mathrm{RCO_2Me}]$$

$$\Longrightarrow [\mathrm{R`CO_2Me}](\Psi\Phi` + \Psi k_{+3} + \Psi`k_{+3}) = [\mathrm{RCO_2Me}](\Psi`\Phi + \Psi`k_{-3} + \Psi k_{-3})$$

$$\Longrightarrow \left.\frac{[\mathrm{R`CO_2Me}]}{[\mathrm{RCO_2Me}]}\right|_{SS} = \frac{\Psi`\Phi+\Psi`k_{-3}+\Psi k_{-3}}{\Psi\Phi`+\Psi k_{+3}+\Psi`k_{+3}} = \frac{\Psi`\Phi+k_{-3}(\Psi+\Psi`)}{\Psi\Phi`+k_{+3}(\Psi+\Psi`)}$$

Taking $\frac{k_{-3}}{k_{+3}} = K_3$ as a factor yields:

$$\left.\frac{[\mathrm{R`CO_2Me}]}{[\mathrm{RCO_2Me}]}\right|_{SS} = K_3\left[\frac{\frac{k_{+3}\Psi`\Phi}{k_{-3}}+k_{+3}(\Psi+\Psi`)}{\Psi\Phi`+k_{+3}(\Psi+\Psi`)}\right]$$

Recognising that $\frac{k_{+3}\Psi`\Phi}{k_{-3}} = \left(\frac{k_{+3}\Psi`\Phi}{k_{-3}\Psi\Phi`}\right)\Psi\Phi` = r_0\Psi\Phi`$ and substituting this expression yields eq. S13:

$$\left.\frac{[\mathrm{R`CO_2Me}]}{[\mathrm{RCO_2Me}]}\right|_{SS} = K_3\left[\frac{r_0\Psi\Phi`+k_{+3}(\Psi+\Psi`)}{\Psi\Phi`+k_{+3}(\Psi+\Psi`)}\right] \quad \text{eq. S13}$$

Examining the form of the denominator and numerator, we see that:

1) the term in [] is always 1 if r_0 = 1, and so $\left.\frac{[\mathrm{R`CO_2Me}]}{[\mathrm{RCO_2Me}]}\right|_{SS} = K_3$ if $r_0$ = 1 (*i.e.,* the concentrations of R`CO_2Me and RCO_2Me are in accordance with the corresponding equilibrium constant).

2) Conversely, if $r_0 \neq 1$ (*i.e.*, coupled reaction is maintained away from equilibrium, kinetic asymmetry is present), the concentrations of R`CO_2Me and RCO_2Me are predicted to deviate from the values predicted by K_3.

This analysis demonstrates that, even though the exchange between RCO_2Me and R`CO_2Me is not *directly* coupled to MeI hydrolysis, because it is part of a cyclic network that contains steps that are, the relative concentrations of these species is perturbed at non-equilibrium steady state and so there is net flux between them.

2. Minor Enantiomer Recycling

Conversion of benzaldehyde (**2**) to the corresponding acyl cyanohydrin (**4**) by reaction with acetoyl cyanide (**3**) can be coupled to the overall hydrolysis of **3** (**Scheme S8a**), leading to a reaction network capable of achieving a non-equilibrium steady state (**Scheme S8b**). Furthermore, because **4** is chiral, by introducing stereoselective catalysts for its formation (cat 1) and hydrolysis (cat 2) it is possible to generate a non-equilibrium steady state in which **4** is significantly enantioenriched. Below we work through the steps required to demonstrate these features.

Note: Throughout, the quoted rate constants are actually "observed" values that include the concentration of the catalyst (*i.e.*, $k_{+1} = k_{+1(\mathrm{real})}[\mathrm{cat\ 1}]$). Compound numbers are as in the main text.

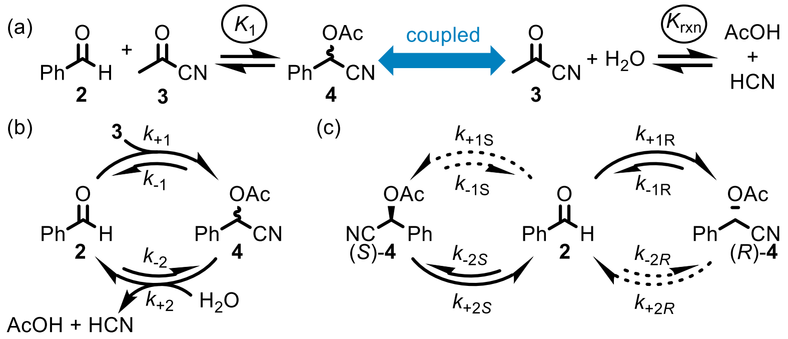

Scheme S8. (a) The coupled reactions in Moberg's MER reaction. (b) simple reaction network showing how the reactions are coupled. (b) The enantiomeric networks in operation the presence of enantioselective catalysts.

2.1 How does coupling acyl cyanohydrin formation to acyl cyanide hydrolysis produce a non-equilibrium steady state?

We can use the simple expression for $\frac{d[\mathbf{4}]}{dt}$ in the simple network (**Scheme S8b**) to generate and expression for $\left.\frac{[\mathbf{4}]}{[2]}\right|_{SS}$:

$$\frac{\mathrm{d}[4]}{\mathrm{dt}} = 0 = k_{+1}[\mathbf{2}][\mathbf{3}] + k_{-2}[2][\mathrm{AcOH}][\mathrm{HCN}] - k_{-1}[\mathbf{4}] - k_{+2}[\mathbf{4}][\mathrm{H_2O}]$$

$$\left.\frac{[\mathbf{4}]}{[2]}\right|_{SS} = \left[\frac{k_{+1}[\mathbf{3}]+k_{-2}[\mathrm{AcOH}][\mathrm{HCN}]}{k_{-1}+k_{+2}[\mathrm{H_2O}]}\right]$$

Taking $[\mathbf{3}]\frac{k_{+1}}{k_{-1}} = [\mathbf{3}]K_1$ as a factor, dividing top and bottom by k_{+2} and substituting $\frac{k_{-1}k_{-2}}{k_{+1}k_{+2}} = \frac{1}{K_{\mathrm{rxn}}}$ yields eq. S14:

$$\left.\frac{[\mathbf{4}]}{[2]}\right|_{SS} = [\mathbf{3}]\mathrm{K}_1\left[\frac{\frac{k_{-1}}{k_{+2}}+[\mathrm{H_2O}]\left\{\frac{1}{K_{\mathrm{rxn}}}\frac{[\mathrm{AcOH}][\mathrm{HCN}]}{[3][\mathrm{H_2O}]}\right\}}{\frac{k_{-1}}{k_{+2}}+[\mathrm{H_2O}]}\right] \qquad \text{eq. S14}$$

From eq. S14 we can see that $\left.\frac{[\mathbf{4}]}{[2]}\right|_{SS} \neq \left.\frac{[\mathbf{4}]}{[2]}\right|_{eq}$ if the coupled reaction (hydrolysis of **4**) is maintained away from equilibrium.

2.2 What conditions must be met to observe an *ee* in using the MER strategy?

Using eq. S14 we can directly write equivalent expressions for $\left.\frac{[\mathbf{4R}]}{[2]}\right|_{SS}$ and $\left.\frac{[\mathbf{4S}]}{[2]}\right|_{SS}$. Dividing $\left.\frac{[\mathbf{4R}]}{[2]}\right|_{SS}$ by $\left.\frac{[\mathbf{4S}]}{[2]}\right|_{SS}$ yields:

$$\left.\frac{[\mathbf{4R}]}{[\mathbf{4S}]}\right|_{SS} = \left[\frac{\frac{k_{-1\mathrm{R}}}{k_{+2\mathrm{R}}}+[\mathrm{H_2O}]\left\{\frac{1}{K_{\mathrm{rxn}}}\frac{[\mathrm{AcOH}][\mathrm{HCN}]}{[3][\mathrm{H_2O}]}\right\}}{\frac{k_{-1\mathrm{R}}}{k_{+2\mathrm{R}}}+[\mathrm{H_2O}]}\right]\left[\frac{\frac{k_{-1\mathrm{S}}}{k_{+2\mathrm{S}}}+[\mathrm{H_2O}]}{\frac{k_{-1\mathrm{S}}}{k_{+2\mathrm{S}}}+[\mathrm{H_2O}]\left\{\frac{1}{K_{\mathrm{rxn}}}\frac{[\mathrm{AcOH}][\mathrm{HCN}]}{[3][\mathrm{H_2O}]}\right\}}\right] \qquad \text{eq. S15}$$

We see from this expression that for any enantiomeric excess (i.e., $\left.\frac{[\mathbf{4R}]}{[\mathbf{4S}]}\right|_{SS} \neq 1$) to be observed at steady state:

1) the coupled reaction must be maintained away from equilibrium;

2) there must be kinetic asymmetry, defined as $\frac{k_{-1S}}{k_{+2S}} \neq \frac{k_{-1R}}{k_{+2R}}$.

2.3 Selectivity in an MER system:

If $[H_2O] >> \frac{k_{-1R}}{k_{+2R}}$ or $\frac{k_{-1S}}{k_{+2S}}$ (reasonable as the reverse of the acylcyanohydrin formation is expected to be slow compared with the rate of hydrolysis) and $[\mathrm{H_2O}]\left\{\frac{1}{K_{rxn}}\frac{[\mathrm{AcOH}][\mathrm{HCN}]}{[\mathbf{3}][\mathrm{H_2O}]}\right\} = \frac{1}{K_{rxn}}\frac{[\mathrm{AcOH}][\mathrm{HCN}]}{[\mathbf{3}]} << \frac{k_{-1R}}{k_{+2R}}$ or $\frac{k_{-1S}}{k_{+2S}}$ (reasonable as the overall hydrolysis reaction is strongly favourable and such reactions are conducted with an excess of the coupled reaction substrates), eq. S15 simplifies to eq. S16, in keeping with previous statements from Moberg and co-workers:[3]

$$\left.\frac{[\mathbf{4R}]}{[\mathbf{4S}]}\right|_{SS} = \left[\frac{\frac{\mathrm{k_{-1R}}}{\mathrm{k_{+2R}}}}{[\mathrm{H_2O}]}\right]\left[\frac{[\mathrm{H_2O}]}{\frac{\mathrm{k_{-1S}}}{\mathrm{k_{+2S}}}}\right] = \frac{k_{-1\mathrm{R}}}{k_{-1\mathrm{S}}}\frac{k_{+2\mathrm{S}}}{k_{+2\mathrm{R}}} = \mathrm{E_1} \times \mathrm{E_2} \qquad \text{eq. S16}$$

Where E_1 and E_2 are the selectivity factors for the acylcyanohydrin forming and hydrolysis catalysts, respectively. Thus, the two catalysts reinforce one another, although the exact value of $\left.\frac{[\mathbf{4R}]}{[\mathbf{4S}]}\right|_{SS}$ depends on the conditions used.

2.4 Iterative MER – reinforcement of *ee* by independent catalysts

If the reactions in the MER cycle are instead run iteratively under effectively irreversible conditions (i.e., very high thermodynamic driving force, short reaction times which means the reverse process is negligible) we can demonstrate how these catalysts reinforce one another.

In step 1, which is the formation of **4** by reaction of **2** with **3** mediated by cat 1, starting from pure **2** the final value of $\frac{[\mathbf{4R}]}{[\mathbf{4S}]}$ is simply $\frac{k_{+1R}}{k_{+1S}} = E_1$.

In step 2, this mixture is subjected to kinetic resolution via the hydrolysis reaction mediated by cat 2. The final ratio at time *t*, $\frac{[\mathbf{4R}]_t}{[\mathbf{4S}]_t}$, is determined by the initial ratio, $\frac{[\mathbf{4R}]_0}{[\mathbf{4S}]_0}$ and the ratio $\frac{k_{+2S}}{k_{+2R}} = E_2$. The rate of change of [**4**R] is:

$$\frac{d[\mathbf{4R}]}{dt} = -k_{+2\mathrm{R}}[\mathbf{4R}][\mathrm{H_2O}]$$

If we assume pseudo-first order kinetics (i.e., $[H_2O]$ is large and thus constant), we can set $k_{+2R}[\mathrm{H_2O}] = k`_{+2R}$ and integrate to get the familiar expression for the change in concentration with time for a first order reaction:

$$\frac{1}{[\mathbf{4R}]}\frac{d[\mathbf{4R}]}{dt} = -k`_{+2\mathrm{R}}$$

Integrating this expression between *t* = 0 and *t* = *t* yields:

$$\mathrm{Ln}\left(\frac{[\mathbf{4R}]_0}{[\mathbf{4R}]_\mathrm{t}}\right) = k`_{+2\mathrm{R}}\mathrm{t}$$

We can rearrange this expression to give a value for $[\mathbf{4R}]_\mathrm{t}$:

$$[\mathbf{4R}]_\mathrm{t} = [\mathbf{4R}]_0\mathrm{e}^{-k`_{+2\mathrm{R}}\mathrm{t}}$$

The same process yields an equivalent expression for $[\mathbf{4S}]_\mathrm{t}$:

$$[\mathbf{4S}]_\mathrm{t} = [\mathbf{4S}]_0\mathrm{e}^{-k`_{+2\mathrm{S}}\mathrm{t}}$$

We can thus write an expression for $\frac{[\mathbf{4R}]_t}{[\mathbf{4S}]_t}$ (eq. S16).

$$\frac{[\mathbf{4R}]_\mathrm{t}}{[\mathbf{4S}]_\mathrm{t}} = \frac{[\mathbf{4R}]_0\mathrm{e}^{-k`_{+2\mathrm{R}}\mathrm{t}}}{[\mathbf{4S}]_0\mathrm{e}^{-k`_{+2\mathrm{S}}\mathrm{t}}} = \frac{[\mathbf{4R}]_0}{[\mathbf{4S}]_0}\mathrm{e}^{\mathrm{t}(k`_{+2\mathrm{S}}-k`_{+2\mathrm{R}})} \qquad \text{eq. S17}$$

We can also generate an expression (eq. S18) for t in terms of the conversion of **4S**, $1 - \frac{[\mathbf{4S}]_t}{[\mathbf{4S}]_0} = x_S$:

$$\mathrm{Ln}\left(\frac{[\mathbf{4S}]_0}{[\mathbf{4S}]_\mathrm{t}}\right) = \mathrm{Ln}\left(\frac{1}{1-x_\mathrm{S}}\right) = k`_{+2\mathrm{S}}\mathrm{t}$$

$$\Rightarrow t = \frac{-1}{k`_{+2S}} \mathrm{Ln}(1 - x_S) \qquad \text{eq. S18}$$

Substituting this value into eq. S17 (note that $\frac{k`_{+2S}}{k`_{+2R}} = \frac{k_{+2S}}{k_{+2R}} = E_2$ as [H_2O] cancels) yields eq. S19:

$$\frac{[\mathbf{4R}]_t}{[\mathbf{4S}]_t} = \frac{[\mathbf{4R}]_0}{[\mathbf{4S}]_0} e^{t(k`_{+2S} - k`_{+2R})} = \frac{[\mathbf{4R}]_0}{[\mathbf{4S}]_0} e^{-\mathrm{Ln}(1-x_S)\frac{k`_{+2S} - k`_{+2R}}{k`_{+2S}}} = \frac{[\mathbf{4R}]_0}{[\mathbf{4S}]_0} e^{-\mathrm{Ln}(1-x_S)\frac{(E_2-1)}{E_2}} \qquad \text{eq. S19}$$

We can also generate an expression for the conversion of **4R**, $1 - \frac{[\mathbf{4R}]_t}{[\mathbf{4R}]_0} = x_R$, in terms of x_S:

$$\mathrm{Ln}\left(\frac{[\mathbf{4R}]_0}{[\mathbf{4R}]_t}\right) = \mathrm{Ln}\left(\frac{1}{1-x_R}\right) = k`_{+2R} t \Rightarrow \left(\frac{1}{1-x_R}\right) = e^{k`_{+2R} t}$$

$$\Rightarrow x_R = \frac{e^{k`_{+2R} t} - 1}{e^{k`_{+2R} t}} = 1 - e^{-k`_{+2R} t} = 1 - e^{\frac{1}{E_2}\mathrm{Ln}(1-x_S)}$$

Using the above equations, taking values of E_1 and E_2 of 10 and setting x_S = 0.8 (i.e., 80% conversion of the minor enantiomer in the second kinetic resolution step), we find that the ratio **2** : (*R*)-**4** : (*S*)-**4** evolves as shown:

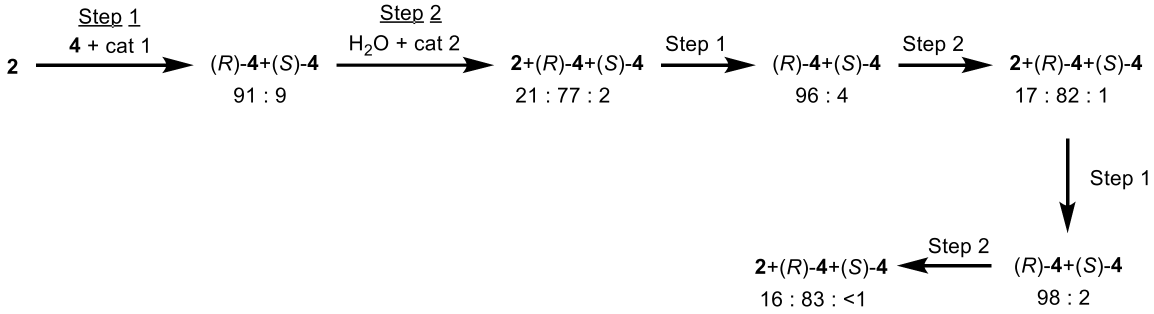

Scheme S9. Evolution of the reaction mixture composition if the acyl cyanohydrin (step 1) and hydrolysis reactions (step 2) are performed iteratively.

3. Operation of catenane 6

In this section we will explore how coupling the base-mediated decomposition of FmocCl to mechanical motion in catenane **6** results in continuous net rotation.

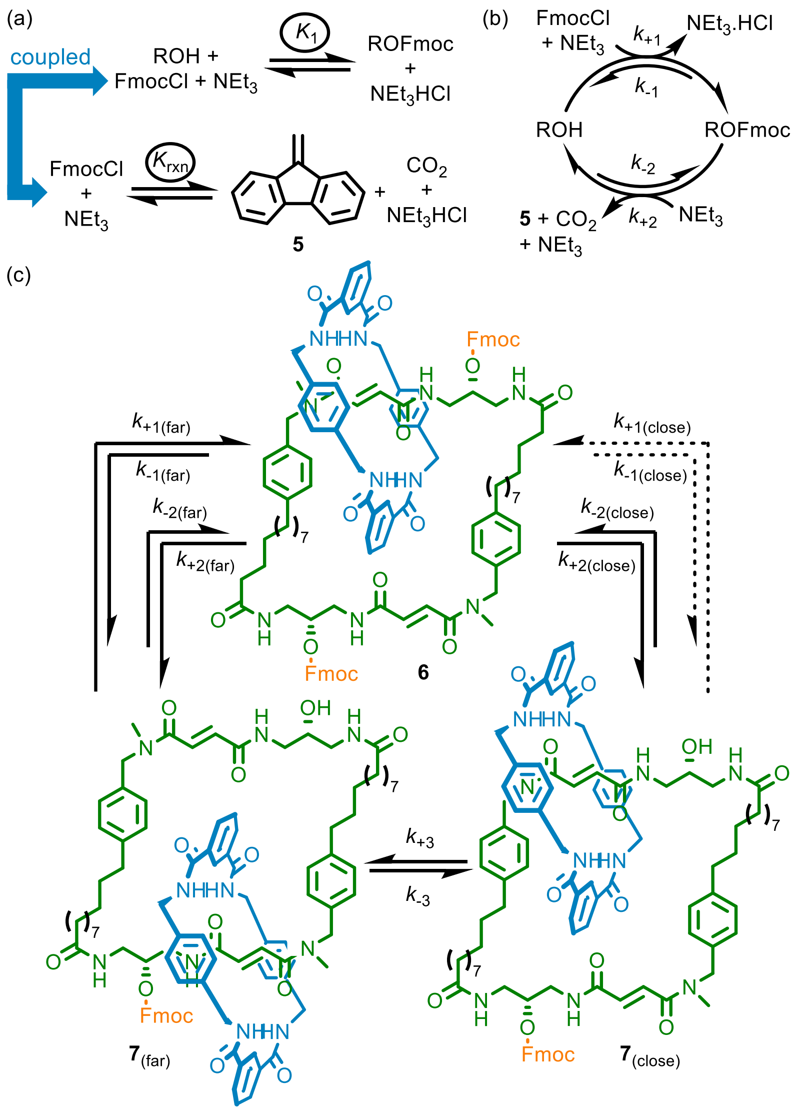

Scheme S10. (a) The reactions that are coupled to generate directional motion in **6**. (b) The network that results from the coupling of these reactions. (c) Schematic representation of the operation of **6** (FmocCl, CO_2, NEt_3 and NEt_3.HCl are omitted for clarity).

3.1 Derivation of the ratchetting constant for catenane 6[2]

Note: throughout, [NEt3] is abbreviated to [B] in the interest of space.

Using eq. S5 starting from **6** and moving clockwise:

$$r_0 = \left(\frac{k_{+2c}[\mathbf{6}][\mathbf{B}]+k_{-1c}[\mathbf{B}.\mathrm{HCl}][\mathbf{6}]}{k_{-2c}[\mathrm{CO_2}][\mathbf{B}][\mathbf{5}][\mathbf{7}_\mathrm{c}]+k_{+1c}[\mathrm{FmocCl}][\mathbf{B}][\mathbf{7}_\mathrm{c}]}\right)\left(\frac{k_{+3}[\mathbf{7}_\mathrm{c}]}{k_{-3}[\mathbf{7}_\mathrm{f}]}\right)\left(\frac{k_{-2f}[\mathrm{CO_2}][\mathbf{B}][\mathbf{5}][\mathbf{7}_\mathrm{f}]+k_{+1f}[\mathrm{FmocCl}][\mathbf{B}][\mathbf{7}_\mathrm{f}]}{k_{+2f}[\mathbf{6}][\mathbf{B}]+k_{-1f}[\mathbf{B}.\mathrm{HCl}][\mathbf{6}]}\right)$$

The concentrations of **6**, **7**$_{c/f}$ and **B** (first denominator, third numerator) cancel, and we can recognise that $\frac{k_{+3}}{k_{-3}} = K_3$ to yield:

$$\mathrm{r}_0 = K_3\left(\frac{k_{+2c}[\mathbf{B}]+k_{-1c}[\mathbf{B}.\mathrm{HCl}]}{k_{-2c}[\mathrm{CO_2}][\mathbf{5}]+k_{+1c}[\mathrm{FmocCl}]}\right)\left(\frac{k_{-2f}[\mathrm{CO_2}][\mathbf{5}]+k_{+1f}[\mathrm{FmocCl}]}{k_{+2f}[\mathbf{B}]+k_{-1f}[\mathbf{B}.\mathrm{HCl}]}\right) = K_3\mathrm{AB}^{-1}$$

Taking $\frac{k_{-1c}[\mathbf{B}.\mathrm{HCl}]}{k_{+1c}[\mathrm{FmocCl}]} = \frac{1}{K_{1c}}\frac{[\mathbf{B}.\mathrm{HCl}]}{[\mathrm{FmocCl}]}$ as a factor from first bracket (A) and dividing top and bottom by k_{-2c} yields:

$$A = \frac{k_{+2c}[\mathbf{B}]+k_{-1c}[\mathbf{B}.\mathrm{HCl}]}{k_{-2c}[\mathrm{CO_2}][\mathbf{5}]+k_{+1c}[\mathrm{FmocCl}]} = \frac{1}{K_{1c}}\frac{[\mathbf{B}.\mathrm{HCl}]}{[\mathrm{FmocCl}]}\left[\frac{\frac{k_{+2c}k_{+1c}}{k_{-2c}k_{-1c}}\frac{[\mathbf{B}][\mathrm{FmocCl}]}{[\mathbf{B}.\mathrm{HCl}]}+\frac{k_{+1c}}{k_{-2c}}[\mathrm{FmocCl}]}{[\mathrm{CO_2}][\mathbf{5}]+\frac{k_{+1c}}{k_{-2c}}[\mathrm{FmocCl}]}\right]$$

Recognizing that $\frac{k_{+2c}k_{+1c}}{k_{-2c}k_{-1c}} = K_1K_2 = K_{rxn}$, and taking $[\mathrm{CO_2}][\mathbf{5}]$ as a factor in first term of numerator yields:

$$A = \frac{1}{K_{1c}}\frac{[\mathbf{B}.\mathrm{HCl}]}{[\mathrm{FmocCl}]}\left[\frac{[\mathrm{CO_2}][\mathbf{5}]\left\{K_{\mathrm{rxn}}\frac{[\mathbf{B}][\mathrm{FmocCl}]}{[\mathbf{B}.\mathrm{HCl}][\mathrm{CO_2}][\mathbf{5}]}\right\}+\frac{k_{+1c}}{k_{-2c}}[\mathrm{FmocCl}]}{[\mathrm{CO_2}][\mathbf{5}]+\frac{k_{+1c}}{k_{-2c}}[\mathrm{FmocCl}]}\right]$$

Apply the same procedure to B yields:

$$B = \frac{1}{K_{1f}}\frac{[\mathbf{B}.\mathrm{HCl}]}{[\mathrm{FmocCl}]}\left[\frac{[\mathrm{CO_2}][5]\left\{K_{\mathrm{rxn}}\frac{[\mathbf{B}][\mathrm{FmocCl}]}{[\mathbf{B}.\mathrm{HCl}][\mathrm{CO_2}][5]}\right\}+\frac{k_{+1f}}{k_{-2f}}[\mathrm{FmocCl}]}{[\mathrm{CO_2}][\mathbf{5}]+\frac{k_{+1f}}{k_{-2f}}[\mathrm{FmocCl}]}\right]$$

Substituting these expressions into r_0 and recognising that ${K_{1f}K_3K_{1c}}^{-1} \equiv 1$ (complete cycle) yields eq. S20:

$$r_0 = \left[\frac{[\mathrm{CO_2}][\mathbf{5}]\left\{K_{\mathrm{rxn}}\frac{[\mathbf{B}][\mathrm{FmocCl}]}{[\mathbf{B}.\mathrm{HCl}][\mathrm{CO_2}][\mathbf{5}]}\right\}+\frac{k_{+1c}}{k_{-2c}}[\mathrm{FmocCl}]}{[\mathrm{CO_2}][\mathbf{5}]+\frac{k_{+1c}}{k_{-2c}}[\mathrm{FmocCl}]}\right]\left[\frac{[\mathrm{CO_2}][\mathbf{5}]+\frac{k_{+1f}}{k_{-2f}}[\mathrm{FmocCl}]}{[\mathrm{CO_2}][\mathbf{5}]\left\{K_{\mathrm{rxn}}\frac{[\mathbf{B}][\mathrm{FmocCl}]}{[\mathbf{B}.\mathrm{HCl}][\mathrm{CO_2}][\mathbf{5}]}\right\}+\frac{k_{+1f}}{k_{-2f}}[\mathrm{FmocCl}]}\right] \quad \text{eq. S20}$$

As previously, $r_0 = 1$ if the coupled reaction is at equilibrium or if there is no kinetic asymmetry ($\frac{k_{+1f}}{k_{-2f}} = \frac{k_{+1c}}{k_{-2c}}$).

Thus, the motor's behavior depends on both the equilibrium constant for the coupled reaction, which depends on ΔG_{rxn} (the properties of the molecules) and the concentrations of the species involved, which do not; the term $e^{-\frac{\Delta G_{\mathrm{rxn}}}{RT}}\frac{[\mathbf{B}][\mathrm{FmocCl}]}{[\mathbf{B}.\mathrm{HCl}][\mathrm{CO_2}][\mathbf{5}]}$ corresponds to the free energy change of the coupled reaction with its components chemostated from their equilibrium values. It should also be obvious from the form of eq. S20 that the motor can display net flux ($r_0 \neq 1$) even if $\Delta G_{\mathrm{rxn}} = 0$, depending on the concentrations of the species involved and can turn in either direction if appropriate values of $[\mathbf{B}]$, $[\mathrm{FmocCl}]$, $[\mathbf{B}.\mathrm{HCl}]$, $[\mathrm{CO_2}]$ and $[\mathbf{5}]$ are taken.

3.2 Relationship between the free energy change associated with mass action and the directionality of 6

We can simplify eq. S20 by recognising that $K_{rxn}\frac{[\mathbf{B}][\mathrm{FmocCl}]}{[\mathbf{B}.\mathrm{HCl}][\mathrm{CO_2}][\mathbf{5}]} = e^{\frac{-\Delta\mu}{RT}}$, where $\Delta\mu$ = the free energy change associated with mass action through one cycle[2] (note: $\Delta\mu$ is negative if the coupled reaction is spontaneous FmocCl→CO_2):

$$r_0 = \left[\frac{[\mathrm{CO_2}][\mathbf{5}]e^{\frac{-\Delta\mu}{RT}} + \frac{k_{+1c}}{k_{-2c}}[\mathrm{FmocCl}]}{[\mathrm{CO_2}][\mathbf{5}] + \frac{k_{+1c}}{k_{-2c}}[\mathrm{FmocCl}]}\right]\left[\frac{[\mathrm{CO_2}][\mathbf{5}] + \frac{k_{+1f}}{k_{-2f}}[\mathrm{FmocCl}]}{[\mathrm{CO_2}][\mathbf{5}]e^{\frac{-\Delta\mu}{RT}} + \frac{k_{+1f}}{k_{-2f}}[\mathrm{FmocCl}]}\right]$$

Dividing through by $[\mathrm{CO_2}][\mathbf{5}]$ yields:

$$r_0 = \left[\frac{e^{\frac{-\Delta\mu}{RT}}+\frac{k_{+1c}}{k_{-2c}}\frac{[\mathrm{FmocCl}]}{[\mathrm{CO_2}][\mathbf{5}]}}{1+\frac{k_{+1c}}{k_{-2c}}\frac{[\mathrm{FmocCl}]}{[\mathrm{CO_2}][\mathbf{5}]}}\right]\left[\frac{1+\frac{k_{+1f}}{k_{-2f}}\frac{[\mathrm{FmocCl}]}{[\mathrm{CO_2}][\mathbf{5}]}}{e^{\frac{-\Delta\mu}{RT}}+\frac{k_{+1f}}{k_{-2f}}\frac{[\mathrm{FmocCl}]}{[\mathrm{CO_2}][\mathbf{5}]}}\right]$$

To rearrange into a useful form, take $e^{\frac{-\Delta\mu}{RT}}$ as a factor of numerator (first bracket) and denominator (second bracket), and divide top and bottom of each bracket by $\frac{k_{+1c}}{k_{-2c}}\frac{[\mathrm{FmocCl}]}{[\mathrm{CO_2}][\mathbf{5}]}$:

$$r_0 = \left[\frac{\frac{k_{-2c}}{k_{+1c}}\frac{[\mathrm{CO_2}][\mathbf{5}]}{[\mathrm{FmocCl}]} + e^{\frac{+\Delta\mu}{RT}}}{\frac{k_{-2c}}{k_{+1c}}\frac{[\mathrm{CO_2}][\mathbf{5}]}{[\mathrm{FmocCl}]} + 1}\right]\left[\frac{\frac{k_{-2f}}{k_{+1f}}\frac{[\mathrm{CO_2}][\mathbf{5}]}{[\mathrm{FmocCl}]} + 1}{\frac{k_{-2f}}{k_{+1f}}\frac{[\mathrm{CO_2}][\mathbf{5}]}{[\mathrm{FmocCl}]} + e^{\frac{+\Delta\mu}{RT}}}\right]$$

If $\frac{k_{-2c}}{k_{+1c}}\frac{[CO_2][\mathbf{5}]}{[FmocCl]} \gg 1$ (and by extension $\frac{k_{-2c}}{k_{+1c}}\frac{[CO_2][\mathbf{5}]}{[FmocCl]} \gg e^{\frac{+\Delta\mu}{RT}}$ given that $\Delta\mu$ is negative) and $\frac{k_{-2f}}{k_{+1f}}\frac{[CO_2][\mathbf{5}]}{[FmocCl]} \ll e^{\frac{+\Delta\mu}{RT}}$:

$$r_0 = \left[\frac{\frac{k_{-2c}[CO_2][5]}{k_{+1c}[FmocCl]}}{\frac{k_{-2c}[CO_2][5]}{k_{+1c}[FmocCl]}}\right]\left[\frac{1}{e^{\frac{+\Delta\mu}{RT}}}\right] = e^{\frac{-\Delta\mu}{RT}} \qquad \text{eq. S21}$$

This situation corresponds to strong gating and the maximum directionality of the catenane motor.

3.3 Relationship between the directionality of 6 and the maximum work that can be performed

If motor **6** is required to do work against a restoring force, the directionality of the motor is modified. The effect of this force can be quite complicated, for example, it may modify the values of the rate constants.[4] However, if we assume these are unchanged, it has previously been shown[2] that *r* will be modified compared to r_0 by a factor of $e^{\frac{w}{RT}}$:

$$r_w = r_0 e^{\frac{w}{RT}}$$

Given that $r_w = 1$ (no net flux) when $w = w_{max}$:

$$e^{\frac{w_{max}}{RT}} = r_0^{-1} \qquad \text{eq. S22}$$

If we substitute the value obtained for r_0 under conditions of strong gating (eq. S21) we find that $w_{max} = \Delta\mu$.

From the above discussion, it should be obvious that the maximum work is not limited by ΔG_{rxn}. Furthermore, if the work done over one reaction cycle takes place over a distance *l*, we see that if $F > \frac{\Delta w_{max}}{l}$, the direction of motor is reversed – as expected, the applied force causes the motor to run backwards.

4. Free energy changes and maximum work of energy ratchets – catenane 11⁺

The operation of catenane **11**⁺ takes place by oscillation of the reaction mixture pH from low (hydrazone gate is labile, ammonium station is protonated) to high (disulfide gate is labile, ammonium station is deprotonated to give an amine), which results in two clockwise half turns of the blue ring (as drawn). Below we dissect these steps to demonstrate that the work done by the motor is not related to the free energy changes of protonation and deprotonation.

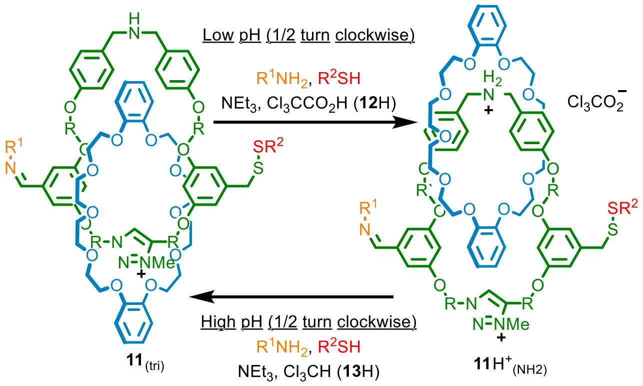

Scheme S11. Operation of catenane motor **11**.

4.1 Maximum work of catenane 11 under conditions of quantitative protonation/deprotonation

If we assume that the protonation/deprotonation and shuttling steps are each essentially quantitative, it should be obvious, because triazolium-**11**⁺ is regenerated and no work is being done, that the free energy change over the full cycle is that of reaction **A**H with **B** (ΔG(AH+B)) – it is simply the free energy of the acid/base reaction.

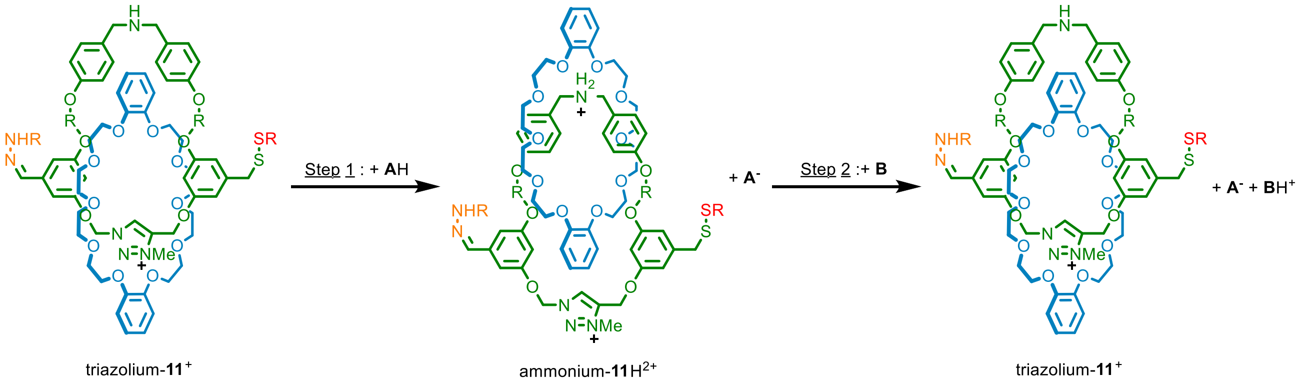

Scheme S12. Operation of **11**⁺ under conditions where both protonation and deprotonation are quantitative, as are the shuttling between the triazolium and the ammonium, and the amine and the triazolium.

The overall free energy change of step 1 (**Scheme S13**), ΔG(step 1) = ΔG(prot) + ΔG(shuttle1), where ΔG(prot) is the free energy of the acid base reaction between the unbound amine and acid AH. Only ΔG(shuttle1) is affected by a restoring force acting against the direction of shuttling. In the absence of a restoring force, ΔG(shuttle1) = ΔG(tri-NH_2) = G(NH_2) – G(tri) (tri = triazolium), the free energy associated with binding of the ring to the ammonium and triazole (tri) respectively. If we require the system to shuttle against a load such that the w_1 work is done shuttling between the triazolium and ammonium stations, the overall free energy change ΔG(shuttle1) = ΔG(tri-NH_2) + w_1. If w_1 = -ΔG(tri-NH_2), the free energy change of shuttling = 0 and thus a 50-50 mixture of the two co-conformations will be produced (i.e., the system is working against its stall force). We assign this value as w_1(max).

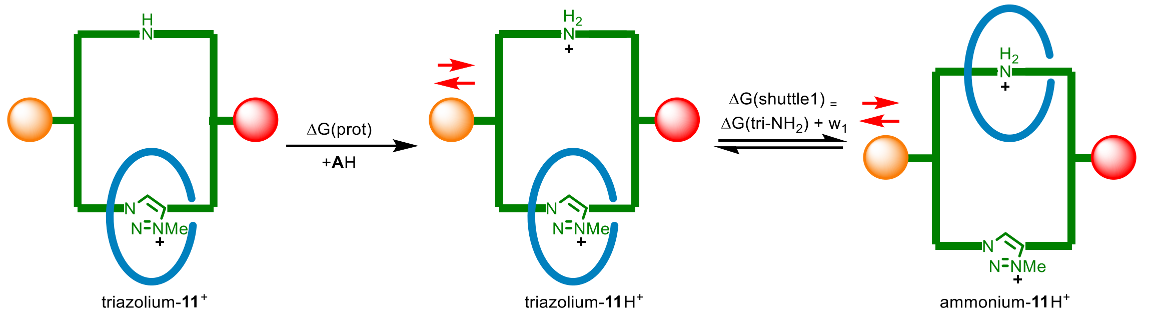

Scheme S13. Step 1 in the operation of **11**$^+$ broken down into a protonation and shuttling step against load. ⇄ indicates that this gate is dynamic (opening and closing) under these conditions..

Using the same approach, we can break step 2 down into a deprotonation event (which is assumed to be quantitative) and a shuttling event. The free energy of deprotonation can be broken down into ΔG(deprot), the free energy of deprotonation for the un-encircled ammonium station, and $\Delta G(NH_2\text{-}NH) = G(NH) - G(NH_2)$, the difference in binding energy for the ring encircling the amine and ammonium respectively. As before, the deprotonation event is not affected by the restoring force but ΔG(shuttle2) is, which leads to an analogous result as above $w_2(max) = -\Delta G(NH\text{-}tri) = G(tri) - G(NH)$.

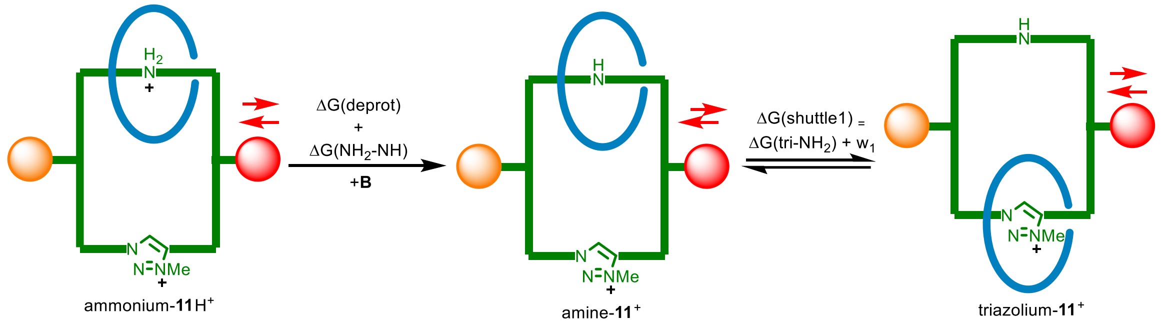

Scheme S14. Step 2 in the operation of **11**$^+$ broken down into a protonation and shuttling step against load. ⇄ indicates that this gate is dynamic (opening and closing) under these conditions.

This very simple treatment under conditions of quantitative protonation/deprotonation demonstrates that the maximum work possible in such systems is a function of the free energy of shuttling, not the free energy associated with protonation/deprotonation.

4.2 Maximum work of catenane 11 under conditions of where protonation/deprotonation are not quantitative

The situation described above is clearly extremely inefficient as the free energy change ΔG(AH+B) would be very large compared with the work done. However, reducing the free energy of protonation/deprotonation results in a more complicated mixture of products in steps 1 and 2 (**Scheme S15**) and a complete discussion lies beyond this manuscript. However, qualitatively, in step 1 it should be obvious that for a weak acid the degree of protonation is enhanced by the binding of the macrocycle to ammonium unit, and hence the degree of protonation is strongly dependent on the restoring force – disfavoring the shuttling in step 1 will also disfavor protonation. Similarly, in step 2, there is a minimum value of ΔG(deprot) required to overcome the additional cost of $\Delta G(NH_2\text{-}NH)$ – if ΔG(deprot) is too small, the macrocycle will remain bound to the ammonium station and no deprotonation or shuttling will take place. Furthermore, any quantitative treatment of such a system would also consider how long each stimulus was applied for – if the pH is varied faster than the rate of shuttling, the motor will never turn even if protonation/deprotonation is taking place. A general quantitative approach for the analysis of such systems taking all these factors into account has been presented by Astumian, which finds that the maximum work such a motor can perform as the conditions are oscillated is a function of the work done on the system by the stimulus.[5]

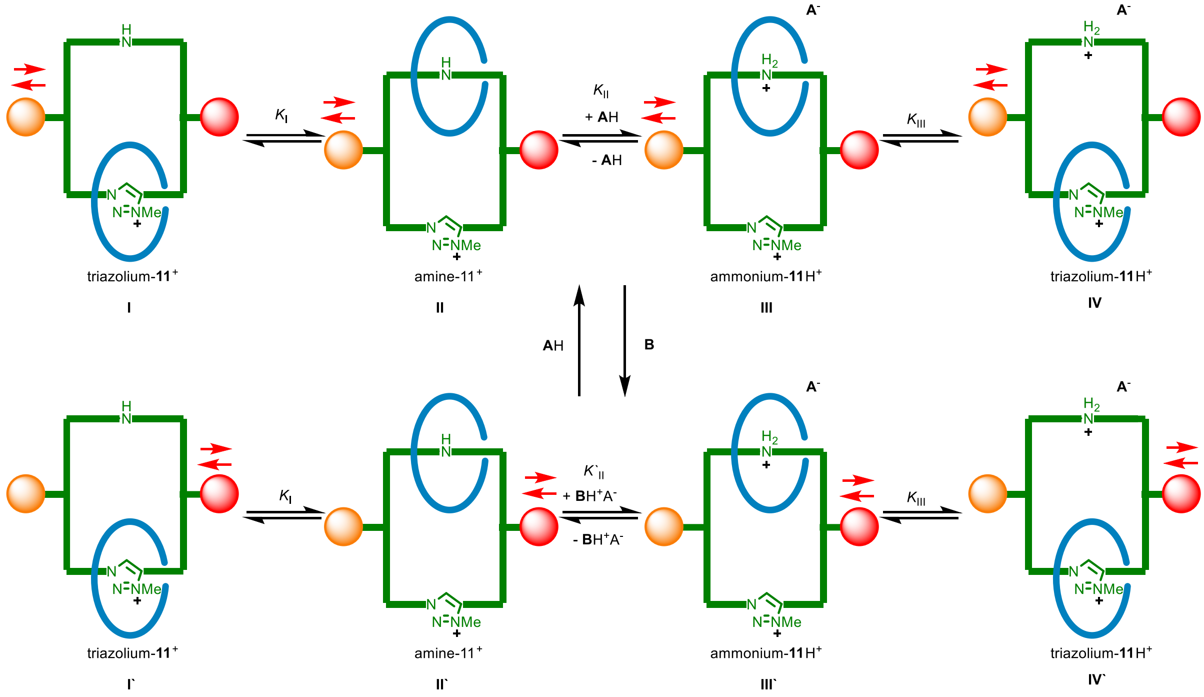

Scheme S15. The complex mixture of products produced as the pH is oscillated if neither protonation nor deprotonation take place quantitatively.

1. Astumian, R. D., Trajectory and Cycle-Based Thermodynamics and Kinetics of Molecular Machines: The Importance of Microscopic Reversibility. *Acc. Chem. Res.* **2018**, *51,* 2653-2661.

2. Astumian, R. D., Kinetic asymmetry allows macromolecular catalysts to drive an information ratchet. *Nat. Commun.* **2019**, *10,* 3837.

3 Fransson, L.; Laurell, A.; Widyan, K.; Wingstrand, E.; Hult, K.; Moberg, C., Minor Enantiomer Recycling-Effect of Two Reinforcing Catalysts on Product Yield and Enantiomeric Excess. *ChemCatChem* **2010**, *2,* 683-693.

4. Astumian, R. D., Irrelevance of the power stroke for the directionality, stopping force, and optimal efficiency of chemically driven molecular machines. *Biophys. J.* **2015**, *108,* 291-303.

5. Feng, Y.; Ovalle, M.; Seale, J. S. W.; Lee, C. K.; Kim, D. J.; Astumian, R. D.; Stoddart, J. F., Molecular Pumps and Motors. *J. Am. Chem. Soc.* **2021**, *143,* 5569-5591.
